# Fu-zi decoction attenuate rheumatoid arthritis *in vivo* and *in vitro* by modulating RANK/RANKL signaling pathway

**DOI:** 10.3389/fphar.2024.1423884

**Published:** 2024-07-23

**Authors:** Zhenzhen Pan, Fangchan Li, Yujie Xu, Huimin Ye, Jiahui Liu, Zhenhua Wang, Changsheng Deng, Jianping Song, Manxue Mei, Changqing Li

**Affiliations:** ^1^ Artemisinin Research Center, Guangzhou University of Chinese Medicine, Guangzhou, China; ^2^ Experimental Teaching Center, Guangxi University of Chinese Medicine, Nanning, China

**Keywords:** Fu-zi decoction, rheumatoid arthritis, osteoclastogenesis, RANK/RANKL, collagen-induced arthritis

## Abstract

**Background:**

Fu-zi decoction (FZD) has a long history of application for treating Rheumatoid arthritis (RA) as a classic formulation. However, its underlying mechanisms have not been fully elucidated. This study aimed to decipher the potential mechanism of FZD in treating RA, with a specific focus on receptor activator of nuclear factor κB/receptor activator of nuclear factor κB ligand (RANK/RANKL) signaling pathway.

**Methods:**

The impact of FZD on RA was investigated in collagen-induced arthritis rats (CIA), and the underlying mechanism was investigated in an osteoclast differentiation cell model. *In vivo*, the antiarthritic effect of FZD at various doses (2.3, 4.6, 9.2 g/kg/day) was evaluated by arthritis index score, paw volume, toe thickness and histopathological examination of inflamed joints. Additionally, the ankle joint tissues were determined with micro-CT and safranin O fast green staining to evaluate synovial hyperplasia and articular cartilage damage. *In vitro*, osteoclast differentiation and maturation were evaluated by TRAP staining in RANKL-induced bone marrow mononuclear cells. The levels of pro- and anti-inflammatory cytokines as well as RANKL and OPG were evaluated by ELISA kits. In addition, Western blotting was used to investigate the effect of FZD on RANK/RANKL pathway activation both *in vivo* and *in vitro*.

**Results:**

FZD significantly diminished the arthritis index score, paw volume, toe thickness and weigh loss in CIA rats, alleviated the pathological joint alterations. Consistent with *in vivo* results, FZD markedly inhibited RANKL-induced osteoclast differentiation by decreasing osteoclast numbers in a dose-dependent manner. Moreover, FZD decreased the levels of pro-inflammatory cytokines IL-6, IL-1β and TNF-α, while increasing anti-inflammatory cytokine IL-10 level both in serum and culture supernatants. Treatment with FZD significantly reduced serum RANKL levels, increased OPG levels, and decreased the RANKL/OPG ratio. In both *in vivo* and *in vitro* settings, FZD downregulated the protein expressions of RANK, RANKL, and c-Fos, while elevating OPG levels, further decreasing the RANKL/OPG ratio.

**Conclusion:**

In conclusion, FZD exerts a therapeutic effect in CIA rats by inhibiting RANK/RANKL-mediated osteoclast differentiation, which suggested that FZD is a promising treatment for RA.

## 1 Introduction

Rheumatoid arthritis (RA) is a challenging autoimmune disease characterized by synovitis, peripheral joint inflammation, and progressive cartilage and bone damage (Di Matteo et al., 2023). Significantly, RA affects approximately 0.46% of the global population, predominantly impacting females aged over 45 (Favalli et al., 2019; Almutairi et al., 2021). The key therapeutic objectives in managing RA emphasize early diagnosis, timely intervention, standardized treatment protocols, and vigilant monitoring to achieve clinical remission, sustain low clinical activity, slow disease progression, minimize disability, and preserve patients’ quality of life (Lin et al., 2020; Cush, 2022). Existing treatment options encompass non-steroidal anti-inflammatory drugs (NSAIDs), glucocorticoids, and disease-modifying anti-rheumatic drugs (DMARDs) (Radu and Bungau, 2021). Some biologic immunosuppressive drugs, such as anti-TNF-α (e.g., Adalimumab and Infliximab) and anti-IL-6 agents (e.g., Tocilizumab, Siltuximab, and Sarilumab), were also utilized to mitigate synovitis and systemic inflammation (Kerschbaumer et al., 2023). However, substantial side effects and high costs limit their sustained clinical utility. Therefore, RA remains an incurable, chronically remitting, and progressively debilitating condition that presents long-term management challenges.

Traditional Chinese Medicine (TCM), especially TCM formula offers a unique strategy to the clinical treatment of RA, particularly during the active stage characterized by moist heat arthralgia spasm syndrome (Wang et al., 2021; Jakobsson et al., 2022). One indispensable TCM formula for RA is Fu-zi decoction (FZD), a formulation dating back over 1,800 years, recorded in Zhang Zhongjing’s Treatise on Febrile Diseases. FZD consists of five key herbs, including Aconiti Lateralis Radix Praeparata (Fuzi), Poria (Fuling), Ginseng Radix Et Rhizoma (Renshen), Atractylodis Macrocephalae Rhizoma (Baizhu), and Paeoniae Radix Alba (Baishao), each chosen for its specific properties within the context of TCM’s cold-heat theory. These herbs collectively aim to alleviate pain, dispel cold pathogens, and restore balance in the body. FZD has been utilized as a complementary and alternative medicine for the clinical treatment of RA in China. Nonetheless, the specific mechanism by which FZD alleviates RA remains unknown, thus hindering its potential for further clinical application.

During the development of RA, there is a significant increase in pro-inflammatory cytokines, leading to redness and pain in the patient’s joints (Krishna Priya et al., 2020; Cheng et al., 2021). Additionally, the inflammatory related mediators induce the production of molecules such as receptor activator of nuclear factor κB ligand (RANKL) to negatively impact cartilage and bone (Kovács et al., 2019). In the advanced stage, the elevated pro-inflammatory factors promote the activity of osteoclasts (OC) within the synovial membrane, consequently resulted in the bone erosion (Wang and He, 2020; Takayanagi, 2021; Yao et al., 2021).

The treatments of RA include the management of inflammation and the bone activity (Zerbini et al., 2017). For the bone activity, the regulation of receptor activator of nuclear factor κB (RANK)/RANK Ligand (RANKL) pathway would benefit the recovery of bone erosion in RA (Tanaka and Ohira, 2018; Liang et al., 2023). RANK/RANKL is known to influence OC activation (Gao et al., 2021; Udagawa et al., 2021; Deng et al., 2023). Furthermore, OC is responsible for the bone absorption in the bone joint, the aberrant increased OC is positively associated with the severity of RA (Auréal et al., 2020).

In our study, we established the classical RA model in rats to evaluate the therapeutic effects of FZD. After treating with the FZD, we observed a notable reduction in inflammation and an improvement in bone damage. This led us to delve deeper into the mechanism, focusing on the inhibitory effects of FZD on specific inflammation markers. We discovered a correlation between the inhibition of FZD and the reduction of these markers in *ex vivo* studies. Recognizing the significance of the RANK/RANKL pathway, we conducted *in vitro* induction assays to elucidate the impact of FZD on OC performance. Encouragingly, FZD demonstrated a reduction in elevated levels of RANKL, effectively modulating the RANK/RANKL pathway. The collective findings from both *in vivo* and *in vitro* studies strongly indicate that FZD holds promise as a therapeutic agent for the treatment of RA.

## 2 Materials and methods

### 2.1 Chemicals and reagents

Bovine type II collagen (CII, 2 mg/mL) and Incomplete Freund’s adjuvant (IFA) were purchased from Chondrex (Redmond, WA, United States). Antibodies against RANK, RANKL, c-Fos and OPG were purchased from ABclonal Technology Co., Ltd. (Wuhan, China). Leflunomide was purchased from Suzhou Changzheng Cinkate Pharmaceutical Corporation (Suzhou, China). TRAP staining kits were purchased from Wuhan Servicebio Technology Co., Ltd. (Wuhan, China) and TRAP assay kits were purchased from Beyotime Biotech Co., Ltd. (Shanghai, China). Recombinant murine sRANK ligand (RANKL) and macrophage colony-stimulating factor (M-CSF) were sourced from PeproTech Technology (Rocky Hill, NJ, United States). ELISA kits for IL-6, IL-10, IL-1β, TNF-α, OPG and RANKL were purchased from Dakewe Biotech Co., Ltd. (Shenzhen, China). Dulbecco’s modified Eagle medium (DMEM), minimum essential medium-α modification (α-MEM) and fetal bovine serum (FBS) was obtained from Gibco (Rockville, United States). Phosphate buffer saline (PBS), penicillin-streptomycin and dimethyl sulfoxide (DMSO) were purchased from Beijing Solarbio Science & Technology Co., Ltd. (Beijing, China).

Reference standards of gallic acid, paeoniflorin, benzoylmesaconine, benzoyl aconitine and atractylenolide III were purchased from the National Institutes for Food and Drug Control (Beijing, China). Reference standards of albiflorin, 1,2,3,4,6-O-pentagalloylglucose and benzoylpaeoniflorin were obtained from Chengdu DeSiTe Biological Technology Co., Ltd. (Chengdu, China). They were prepared at a proper concentration in methanol. HPLC-grade acetonitrile and phosphoric acid were obtained from Fisher Scientific (Loughborough, UK). Ultrapure water was prepared by the Milli-Q plus water purification system.

### 2.2 Animals and cells

Sprague-Dawley female rats (6–8 weeks old, weighing 180–220 g) and C57BL/6 male mice (4–6 weeks old, weighing 12–18 g) were purchased from Hunan SJA Laboratory Animal Co., Ltd. (Hunan, China) and the animal license number is SCXK (Xiang) 2019-0014. The rats were raised at a suitable temperature (25°C) with a 12 h light/dark cycle. All animal experiments were approved by Institutional Animal Welfare and Ethical Committee of Guangxi University of Chinese Medicine (Ethical Approval Number: DW20230517-085).

Bone marrow mononuclear cells were separated from the tibias and femurs of 4–6 weeks-old C57BL/6 mice. Cells were cultured in α-MEM containing 10% FBS and 1% penicillin/streptomycin supplemented with M-CSF (30 ng/mL) for 4 days. The adherent cells left at the bottom of the culture dish were considered as BMMs (Li Y. et al., 2022).

### 2.3 Preparation of FZD

FZD contains five traditional herbs (Chen et al., 2022), including 15 g of Aconiti Lateralis Radix Praeparata (*Aconitum carmichaeli* Debx.), 9 g of Poria (*Poria cocos* (Schw.) Wolf), 6 g of Ginseng Radix Et Rhizoma (*Panax ginseng* C. A. Mey.), 12 g of Atractylodis Macrocephalae Rhizoma (*Atractylodes macrocephala* Koidz.) and 9 g of Paeoniae Radix Alba (*Paeonia lactiflora Pall.*). All herbs were soaked in water for 30 min and boiled twice with 10 times water (an hour each time). For experiments *in vivo*, the filtrate solutions were collected and condensed to 0.78 g/g (1 g of extract is equivalent to 0.78 g of the original prescription) as stock solution and stored at 4°C. This stock solution was diluted with distilled water to the proper concentration before use. For HPLC fingerprint analysis and experiments *in vitro*, the filtered solutions were evaporated and followed freeze-dried by freeze-drier, yielded dried powder and stored in a dryer for future use.

### 2.4 High-performance liquid chromatography (HPLC) fingerprinting of FZD

HPLC fingerprint analysis was operated on Shimadzu LC2030 high-performance liquid chromatograph, and all samples were separated on a Thermo Acclaim™ 120 C_18_ column (250 × 4.6 mm, 5 μm). The mobile phase was composed of acetonitrile (A) and 0.05% phosphoric acid-water (B). The condition used for the gradient program was developed as follows: 0–8 min, 5%A; 8–33 min, 5–16%A; 33–40 min, 16%A; 40–58 min, 16–18%A; 58–65 min, 18–25%A; 65–75 min, 25%A; 75–80 min, 25–35%A; 80–95 min, 35–46%A; 95–108 min, 46–90%A. The flow rate was 0.8 mL/min, and the column temperature was 30°C. The detection wavelength was set at 230 nm, and the injection volume of each sample and standard solution was 10 µL.

15 batches of each dried powder were passed through a sieve (mesh 80), and 0.5 g of powder that was accurately weighed and refluxed with 5 mL of methanol-0.05% hydrochloric acid at 70°C for 0.5 h. The extraction was filtrated through a 0.45 µm membrane, 10 µL of the obtained solution was injected into the HPLC system for analysis. Similarity Evaluation System for Chromatography Fingerprint of Traditional Chinese Medicine (version 2012A, Chinese Pharmacopoeia Commission, Beijing, China) was used to establish the fingerprint and analyze the similarity by importing the chromatograms of 15 batches of FZD.

### 2.5 FZD treatment on CIA model

The collagen-induced arthritis (CIA) model was established based on the previous reports (Ba et al., 2021). Bovine type II collagen was emulsified with equal volume IFA on the ice. Except for the control group (Control, n = 10), the rats received a subcutaneous injection of 200 µL emulsion prior prepared at the base of the tail, and another 100 µL emulsion was injected on day 7 in the same manner for the boost immunization. Control group rats were injected with an equal volume of normal saline instead of emulsion. To evaluate the severity of CIA, the condition of the paws was monitored and estimated every 4 days on a scale of 0–4, as shown in Table 1. The arthritis index score was calculated using the cumulative score for all four paws of each rat, with a maximum value of 16 per animal. The severity of arthritis was assessed by visual observation by three independent observers. On the 12th day of modeling, the successfully modeled rats with the arthritis indexes over than three were randomly divided into five groups: model control group (Model, n = 10), Leflunomide-treated group (LEF, n = 10), FZD-Low/Medium/High groups (n = 10 per group). Based on the recommended clinical dosage of FZD at 51 g/70 kg/day for patients with RA, the dosage for rats (with an average weight of 200 g) was calculated using a body surface area conversion factor of 6.3 (Nair et al., 2018). Based on the calculation results, the doses of FZD in the low, medium, and high dose groups were determined to be 2.3, 4.6, and 9.2 g/kg/day, respectively. These dosages corresponded to 0.5, 1, and 2 times the recommended daily dose for RA patients. The Leflunomide-treated group was treated with Leflunomide (1.8 mg/kg/day). In addition, both the control rats and model rats were given normal saline (10 mL/kg/day). All rats were administered once daily for 30 days. During the treatment period, the paw volume of the rats was measured every 7 days with a PV-200 Toe volume measuring instrument (TECHMAN Soft, Chengdu, China). At the same time, toe thickness was measured using a vernier caliper. The body weight of the rats was monitored with 0.1 g precision balance every 4 days.

**TABLE 1:** Scoring criteria.

Score	Symptom
0	no swelling and erythema
1	mild but definite redness and swelling of the ankle or wrist, or apparent redness and swelling limited to individual digits
2	local toe joint and dorsum pedis are affected
3	severe redness and swelling of the entire paw, including digits
4	the entire paw affected (maximal erythema and swelling) with motor dysfunction

At the experimental endpoint, the rats were sacrificed, blood samples were obtained, and the major organs were harvested and weighed from each group. The levels of serum IL-6, IL-1β, TNF-α, IL-10, RANKL and OPG were determined by commercially available ELISA kits. The left ankle joints were fixed in 10% neutral-buffered formalin for micro computed tomography imaging (micro-CT). Histopathological examination of the major organs and synovium of ankle/knee joints (left) were conducted. Besides, the ankle joint tissues were stained with safranin O fast green (Safranin O) for evaluation of synovial hyperplasia and articular cartilage damage. A collection of histopathological sections was obtained using the 3D HISTECH Pannoramic 250 imaging system. Blood samples were collected and centrifuged at 3500 *g* for 15 min (4°C), and the serum was stored at −80°C until use.

$$\text{The organ coefficient was calculated using the following formula}:\text{Organ coefficient }\left(\%\right)=\text{Weight of the organ}/\text{Body weight}\times 100\%$$

### 2.6 Cell viability assay

The cytotoxicity of FZD on BMMs was evaluated with a standard MTT assay. Briefly, the BMMs were seeded and cultured in 96-well plates, with a density of 5 × 10^3^ cells per well (30 ng/mL M-CSF) overnight. Then, cells were added with indicated concentration of FZD (0, 50, 100, 125, 250, 500, 1000, 2000, 4,000 μg/mL) for 24 or 48 h, respectively. Afterward, 20 µL of MTT solution (5 mg/mL in PBS) was added to each well and the cells were incubated for another 4 h. Subsequently, the medium was removed, and the cells were dissolved in 200 µL of DMSO per well. The absorbance was measured by using a microplate reader (Multiskan GO, Thermo, USA) at 490 nm. Cell viability was calculated with the formula:

$$\text{Cell viability }\left(\%\right)=\left[\left(A_{T}-A_{B}\right)/\left(A_{C}-A_{B}\right)\right]\times 100\%$$

Where A_T_, A_C_, and A_B_ represent the absorbance of the treated cells, the untreated cells, and the blank culture media, respectively.

### 2.7 *In vitro* osteoclast differentiation and tartrate-resistant acid phosphatase staining

For osteoclast differentiation, BMMs cells were inoculated in 48-well plates (2×10^4^ cells/well) in complete α-MEM supplemented with 30 ng/mL M-CSF, 100 ng/mL RANKL, and different concentrations of FZD (0, 500, 1000 or 2000 μg/mL) for 7 days. The culture medium was replaced every other day. On the seventh day, the supernatant of each well was collected for ELISA assays. For TRAP staining, the cells were then washed twice with PBS, fixed with 4% paraformaldehyde for 20 min, and stained for TRAP according to the manufacturer’s instruction. TRAP-positive multinucleated (nuclei≥3) cells were considered osteoclasts, and counted in randomly selected visual fields in different areas of each well under a microscope (DMI3000 B, Leica, Germany). For the TRAP activity assay, the cells were collected and lysed, and then the supernatant of the cell lysates was quantified for the TRAP activity by using Tartrate Resistant Acid Phosphatase Assay Kit.

### 2.8 Enzyme-linked immunosorbent assay

The levels of IL-6, IL-1β, TNF-α and IL-10 in the culture supernatants and serum were determined using ELISA kits respectively. All the operations were based on the manufacturer’s protocols. The optical density was measured at 450 nm and the concentration was calculated from a standard curve.

### 2.9 The induction of *in vitro* OC for western blotting

The BMMs cells were seeded into 6-well plates at a density of 4×10^5^ cells per well with M-CSF (30 ng/mL) and were incubated for 24 h before use. Then the cells were treated with FZD (0, 500, 1000 or 2000 μg/mL) and 100 ng/mL RANKL for 5 days. The cells were then collected and prepared the samples after a thorough wash with cold PBS.

### 2.10 Western blotting

The protein expression levels of RANK, RANKL, OPG and c-Fos in ankle joint tissues or BMMs cells were detected by Western blotting assay. Briefly, ankle joint tissues and cells were independently homogenized in RIPA buffer containing protease inhibitors. Total proteins from ankle joint tissues and cells were quantitated using BCA protein quantification kits according to the manufacturer’s instructions. The proteins were then separated using 10% SDS-PAGE and blotted onto polyvinylidene fluoride (PVDF) membranes. The protein-bearing membranes were blocked in protein free rapid blocking buffer for 15 min and incubated overnight at 4°C with primary antibodies including those recognizing RANK, RANKL, OPG and c-Fos. GAPDH and β-actin were used as internal references. Then the membranes were then hybridized with horseradish peroxidase (HRP)-conjugated secondary antibodies for 1 h. The immunoreactive protein bands were visualized using an enhanced chemiluminescence (ECL) system. ImageJ was used to quantify band intensities. All experiments were performed in biological triplicates (n = 3), and data were representative of three independent experiments.

### 2.11 Statistical analysis

Data analyses were performed using GraphPad Prism 9.0. All data were depicted as the mean ± standard deviation (SD). Statistical differences between the groups were determined by one-way ANOVA. *P*< 0.05 was considered significant.

## 3 Results

### 3.1 HPLC fingerprints of FZD

**FIGURE 1:**
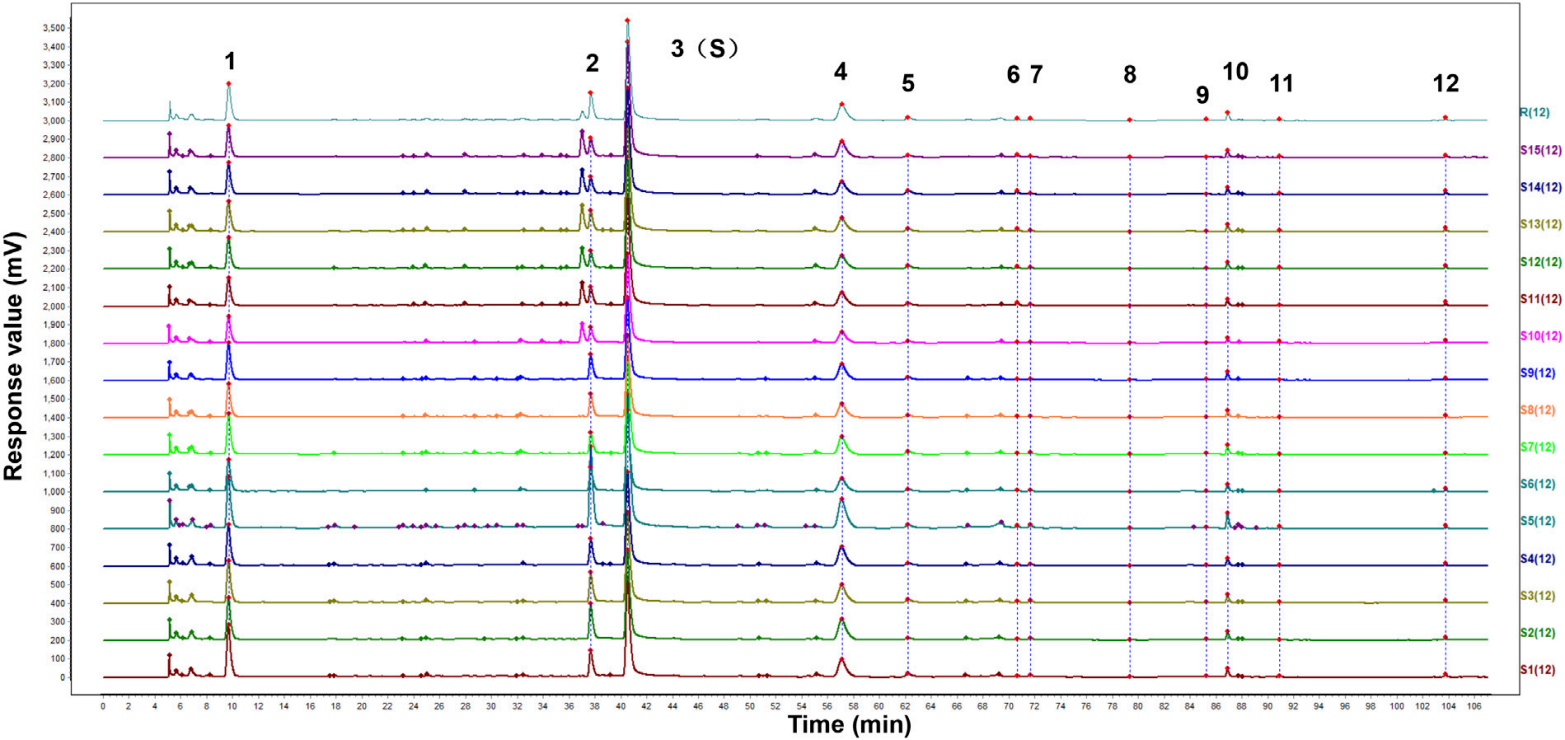
HPLC fingerprints of 15 batches of FZD. The X-axis represents the retention time (t/min) and the Y-axis represents the response value (mV). The peaks 1-12 were the main common peaks. (1 gallic acid; 2 albiflorin; 3 paeoniflorin; 5 1,2,3,4,6-O-pentagalloylglucose; 6 benzoylmesaconine; 8 benzoyl aconitine; 10 benzoylpaeoniflorin; 12 atractylenolide III).

The HPLC fingerprints from 15 batches of FZD were established by Similarity Evaluation System for Chromatography Fingerprint of Traditional Chinese Medicine. Peaks that were existed in all sample chromatograms with reasonable heights and good resolutions were assigned as common peaks. The time window was set to 0.5 s, and the calibration method was multipoint calibration. The reference chromatogram fingerprint was generated by using the average method. As shown in Figure 1, there were 12 distinct common peaks in the HPLC fingerprints, eight of which (peaks 1, 2, 3, 5, 6, 8, 10 and 12) were identified as gallic acid, albiflorin, paeoniflorin, 1,2,3,4,6-O-pentagalloylglucose, benzoylmesaconine, benzoyl aconitine, benzoylpaeoniflorin and atractylenolide III, respectively, by comparing retention times with the standard compounds.

The similarity of chromatographic fingerprint data is indicated by the correlation coefficient. The value of the correlation coefficient is close to 1.0, indicating that the different samples there have high similarity. On the contrary, a low correlation coefficient indicates a poor mathematical quality for identifying the relationship between different samples. As shown in Table 2, the similarity of the samples was larger than 0.97, indicating that FZD had good similarity and shared similar chemical components. The results confirmed that the fingerprints established in this study were reliable in assessing the quality of FZD.

**TABLE 2:** The similarity values for 15 batches of FZD.

Sample	Similarity value
FZD 1	0.993
FZD 2	0.990
FZD 3	0.996
FZD 4	0.997
FZD 5	0.974
FZD 6	1.000
FZD 7	0.998
FZD 8	1.000
FZD 9	0.996
FZD 10	0.995
FZD 11	0.991
FZD 12	0.992
FZD 13	0.994
FZD 14	0.993
FZD 15	0.993

### 3.2 FZD show efficacy in reducing arthritis features/indices

**FIGURE 2:**
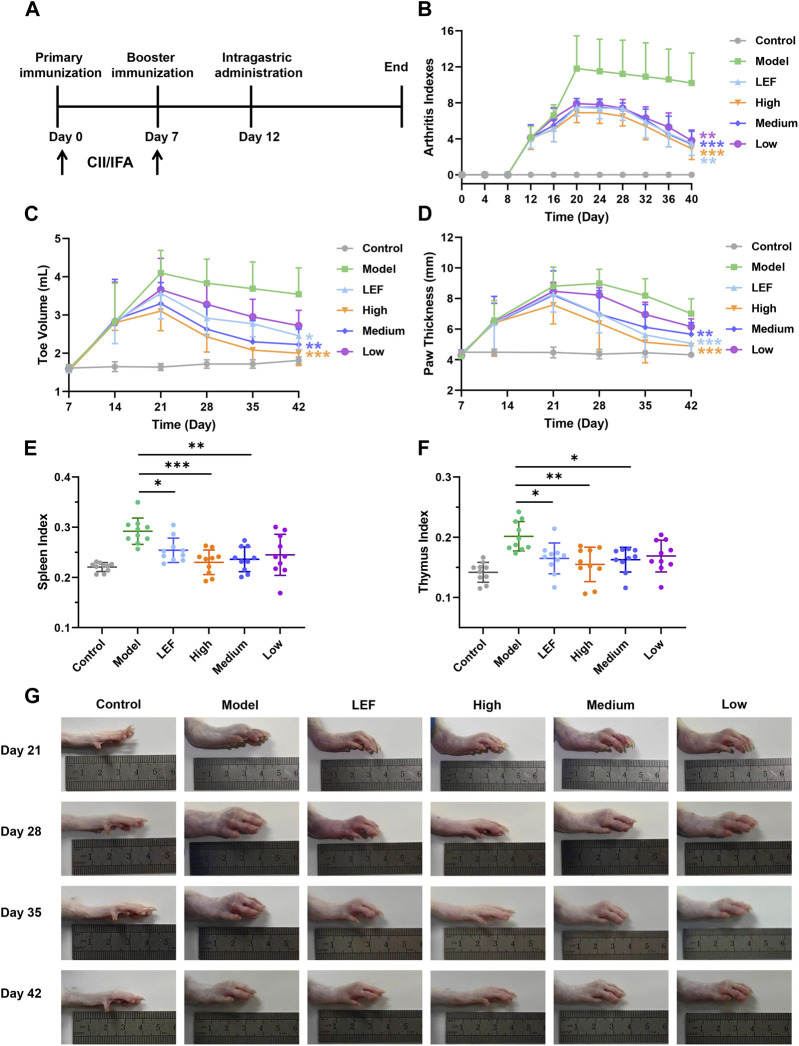
FZD ameliorated arthritis severity in CIA rats (Dosages, High group = 9.2, Medium group = 4.6, Low group = 2.3, g/kg/day). **(A)** Experimental scheme of the analysis of CIA rats. **(B)** Arthritis index score. **(C)** Changes in the toe volume were assessed in each group. **(D)** Changes in paw thickness. **(E)** Spleen index and Thymus index **(F)**. **(G)** Representative images of the swollen left hind paws in each group. Values are presented as the mean ± SD, n = 10, *P< 0.05, **P< 0.01, ***P< 0.001, compared with the model.

We developed a CIA model, as detailed in Figure 2A, rats received a subcutaneous injection of a 200 µL emulsion at the base of the tail, followed by another 100 µL emulsion on day 7 for boost immunization. As shown in Figure 2B, the arthritis index exhibited a sharp increase on day 8, reaching its maximum value on day 20. Notably, the treated groups, irrespective of dose, displayed lower increases. FZD significantly reduced the arthritis index between days 20–40 in the 2.3 g/kg/day and 9.2 g/kg/day (*P <* 0.05). Similarly, as depicted in Figure 2C toe volume increased from day 7, peaking at 4 mL on day 21. The increase of toe volume was because of the swelling in rat toes. Fortunately, even at a low dose, FZD administration was able to reduce toe swelling and ultimately decrease toe volume. The impact of FZD in the medium and high dose groups also surpassed the dramatic swelling observed in model group. Paw thickness, a critical indicator, exhibited high values in the model groups on day 21, with minor decreases over time due to self-immunity. In contrast, FZD at a high dose significantly reduced paw edema from value 8 on day 21 to 4.5 on day 42, approaching healthy control levels. The medium group showed a significant decrease in paw thickness over time, particularly on day 42 (Figure 2D). The spleen index, reflecting the strength of immune response, increased in the model group (Figure 2E), indicating significant inflammation. Treatment, which encompassed both Leflunomide-therapeutics and FZD at varying doses, led to a reduction in the spleen index. Moreover, thymus index, an indicative of thymus reaction to inflammatory pathogens, increased to 0.25 in the model group compared to the control group (Figure 2F). However, when treating with FZD, this parameter decreased to 0.15, suggesting a reduction in thymus reaction. In Figure 2G, representative photos of the left ankle joint reveal significant findings throughout the experimental timeline. On day 21, observable swelling in the model group, and rats in this group experienced impaired mobility and reduced food intake due to pain. Contrastingly, FZD-treated rats exhibited minor swelling. On day 28, swelling in the model group still shown increased trend, but FZD treatment suppressed this increase. The swelling conditions improved by day 42 due to self-immunization, while residual swelling still persisted. Differently, the swelling ankle in the high dose of FZD exposure back to nearly normal.

### 3.3 FZD exposure increase knee and ankle joint pathology and cartilage integrity

**FIGURE 3:**
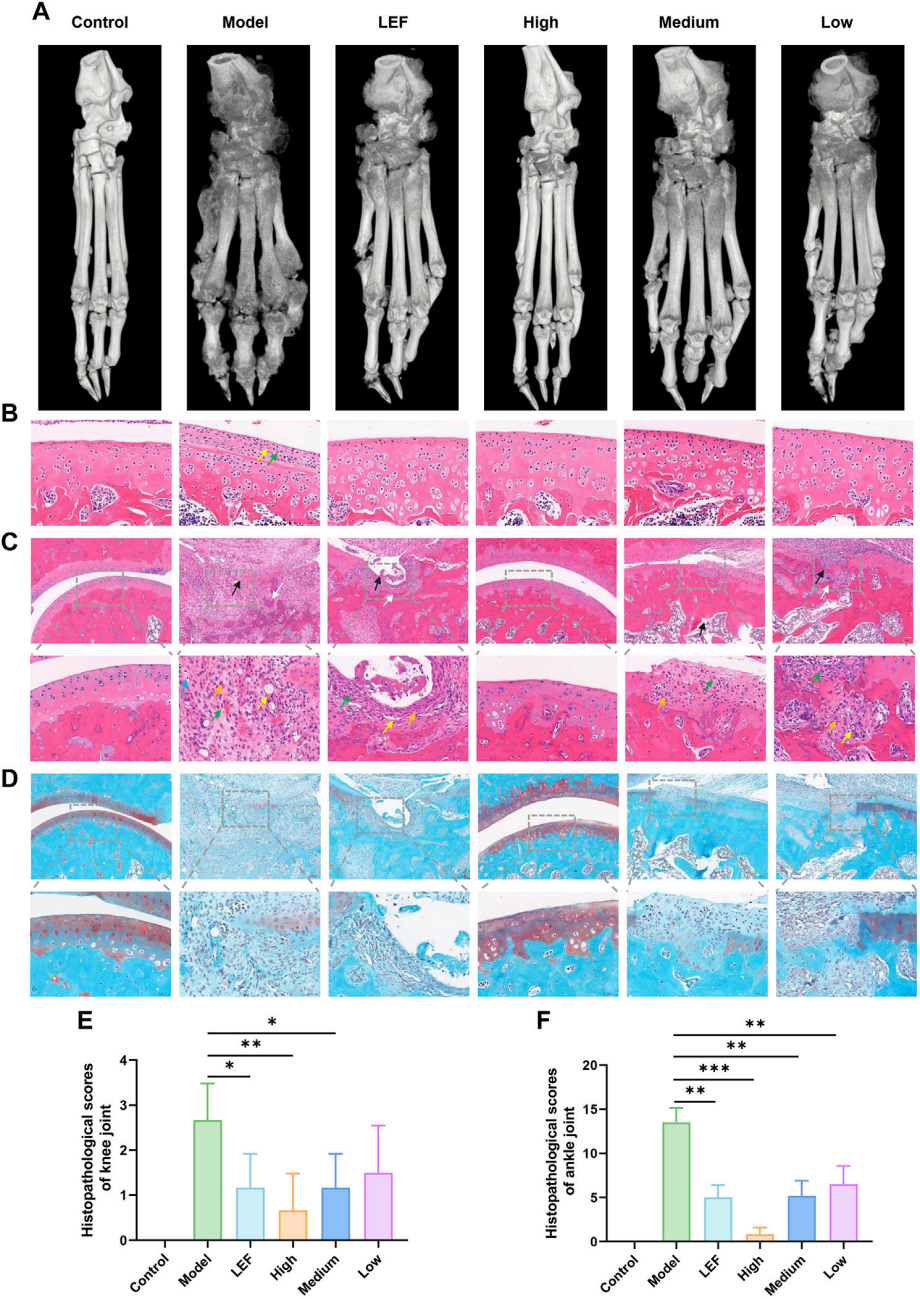
FZD improved histopathological inflammation in CIA rats (Dosages, High group = 9.2, Medium group = 4.6, Low group = 2.3, g/kg/day). **(A)** Representative photographs of micro-CT. **(B)** HE staining of synovial tissue of knee (×400; yellow arrow: fibroplasia; green arrow: plasma cell). **(C)** HE staining of ankle joint synovia (above: ×100; below: ×400; black arrow: cartilage defect; white arrow: trabecular necrosis; blue arrow: leukomonocyte; orange arrow: fibroplasia; green arrow: macrophagocyte; yellow arrow: new blood capillary). **(D)** safranin O fast green staining of ankle joint synovium (above: ×100; below: ×400). **(E)** Histopathological scores of knee joint. **(F)** Histopathological scores of ankle joint.

Micro-CT analysis (Figure 3A) highlighted increased toe bone joint space in the model group, indicating severe bone erosion. Additionally, a rough bone surface suggested decreased bone density and an increased risk of fracture. Both the FZD-treated groups and the Leflunomide-treated group exhibited improvement in bone erosion and density loss, with the high dose showing the most significant efficacy. Figures 3B, C present histopathological findings of knee and ankle joint synovium, along with corresponding cartilage assessments. In knee joint synovium pathology (Figure 3B), the model group exhibited minimal inflammatory cell infiltration and fibrous tissue proliferation, but no significant cartilage defects or chondrocyte necrosis. Formulation-treated groups showed no noteworthy changes. In ankle joint synovium pathology (Figure 3C), the healthy control group displayed intact cartilage with no pathological changes. In contrast, the model and Leflunomide-treated groups showed extensive cartilage defects, thinning, chondrocyte necrosis, and trabecular bone necrosis. The high dose FZD group maintained intact cartilage, while the medium dose showed reduced magnitude of observed changes. The low dose group displayed similar findings to the medium dose. Figure 3D depicted safranin O fast green staining, showing uniform thickness and a complete tide line in the control group. The model group exhibited complete cartilage loss, while the Leflunomide-treated group showed partial loss and uneven staining. High dose FZD maintained a healthy cartilage structure, while the medium and low doses displayed mild to moderate cartilage damage. By evaluating the histopathological scoring results of knee and ankle joints, the impact of FZD on RA can be more precisely ascertained (Figures 3E, F).

### 3.4 FZD modulated inflammatory cytokines

**FIGURE 4:**
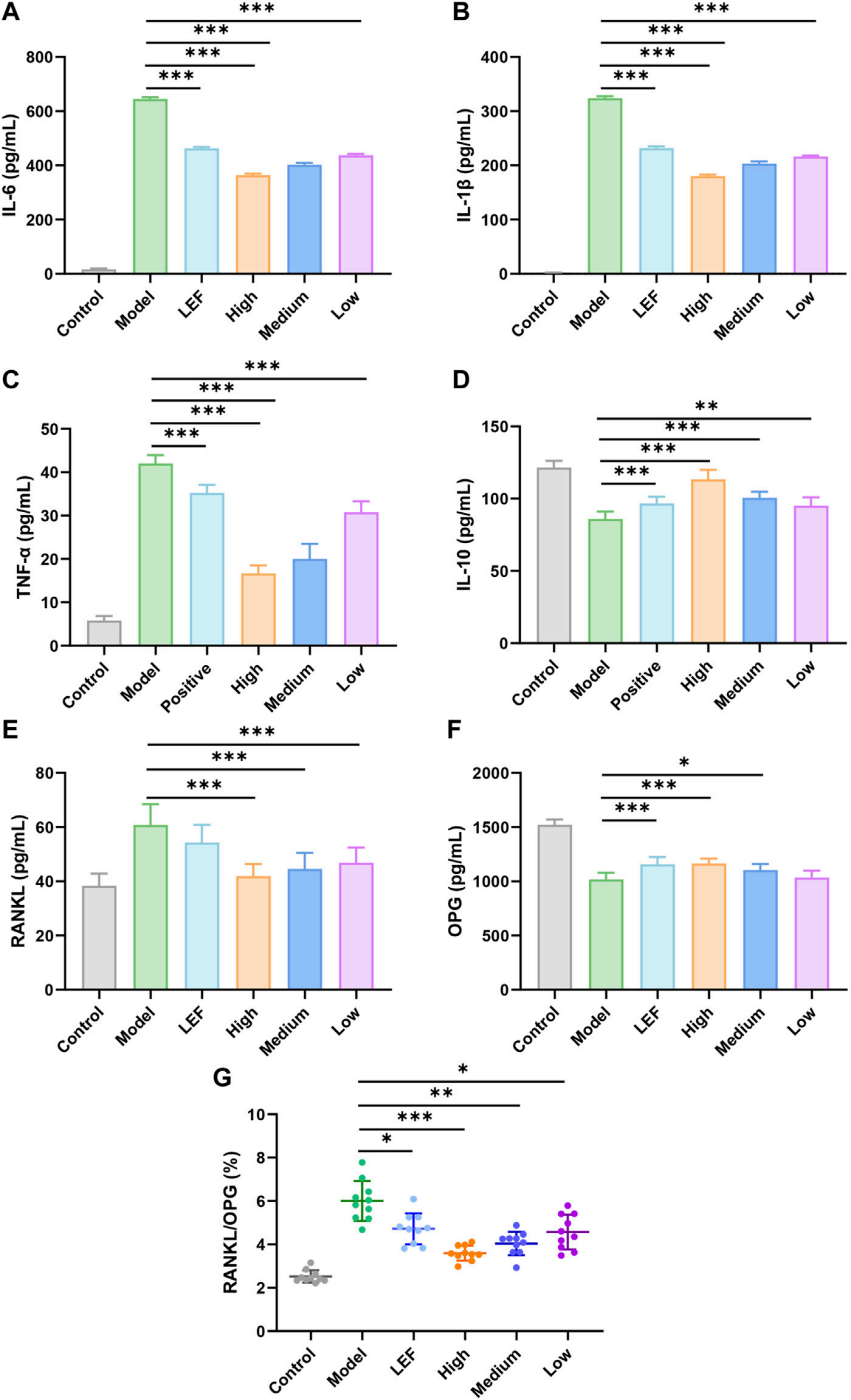
Effects of FZD on the expression of IL-6 **(A)**, IL-1β **(B)**, TNF-α **(C)**, IL-10 **(D)**, RANKL **(E)**, OPG **(F)**, RANKL/OPG **(G)** in serum (Dosages, High group = 9.2, Medium group = 4.6, Low group = 2.3, g/kg/day). The results are expressed as mean ± SD, n = 10, *P< 0.05, **P< 0.01, ***P< 0.001, compared with the model.

IL-6 is a classic marker of inflammation, the stark elevation of IL-6 levels in the model group portrayed a vivid portrait of heightened inflammatory cascades occurring. However, the significant decreases in the FZD exposure with three doses were observed. Among them, the high dose exhibited the most significant downregulations effects (Figure 4A). Similar to IL-6, the model group showed a robust surge in IL-1β levels, unveiling the intensity of inflammatory processes experienced by model rats. With FZD exposure, IL-1β decreased to approximately 100 pg/mL (Figure 4B). The TNF-α within the model group significantly surge, however, the statistical decrease of TNF-α was found in the FZD exposure group. The decrease of TNF-α was a testament of migration to the vigorous inflammatory stimuli encountered by the rats (Figure 4C). Furthermore, we examined the levels of IL-10, where a discernible reduction within the FZD treated group was observed, suggesting a potential inflammation suppression of FZD (Figure 4D). RANKL is an important promotion factor for the development of bone-associated disease including RA. Excessive RANKL expression may disrupt the balance between osteoblasts and osteoclasts, leading to compromised bone formation, increased bone resorption, and ultimately resulting in decreased joint bone mass, bone erosion, and bone damage. The impact of FZD on RANKL illustrated in Figure 4E, the model group showing a substantial upswing of RANKL suggested heightened activity in bone absorption. FZD led to a downregulation of RANKL expression, indicating an improvement in bone erosion activity. Conversely, OPG is a factor reflecting the bone build or bone intensity recovery and the inflammatory factors diminished OPG in RA microenvironment. Figure 4F suggests that the model group exhibited a diminished presence of OPG, implying the damage to bone-built within the RA inflammatory microenvironment. To further confirm the efficacy of FZD on bone activity, we calculated the ratio of RANKL/OPG. The model group manifests a pronounced elevation in the RANKL/OPG ratio, indicative of a disrupted equilibrium favoring the process of bone resorption within the inflammatory milieu (Figure 4G). But in the treated groups, we found that FZD decrease the bone erosion in CIA rats, being an anti-bone erosion mechanism and providing explanations for Figure 3 results. Of note, exposure to FZD resulted in significant decreases the RANKL/OPG ratios, with the high dose exhibiting the most pronounced downregulatory effects.

### 3.5 Preliminary evaluation of the treatment safety

**FIGURE 5:**
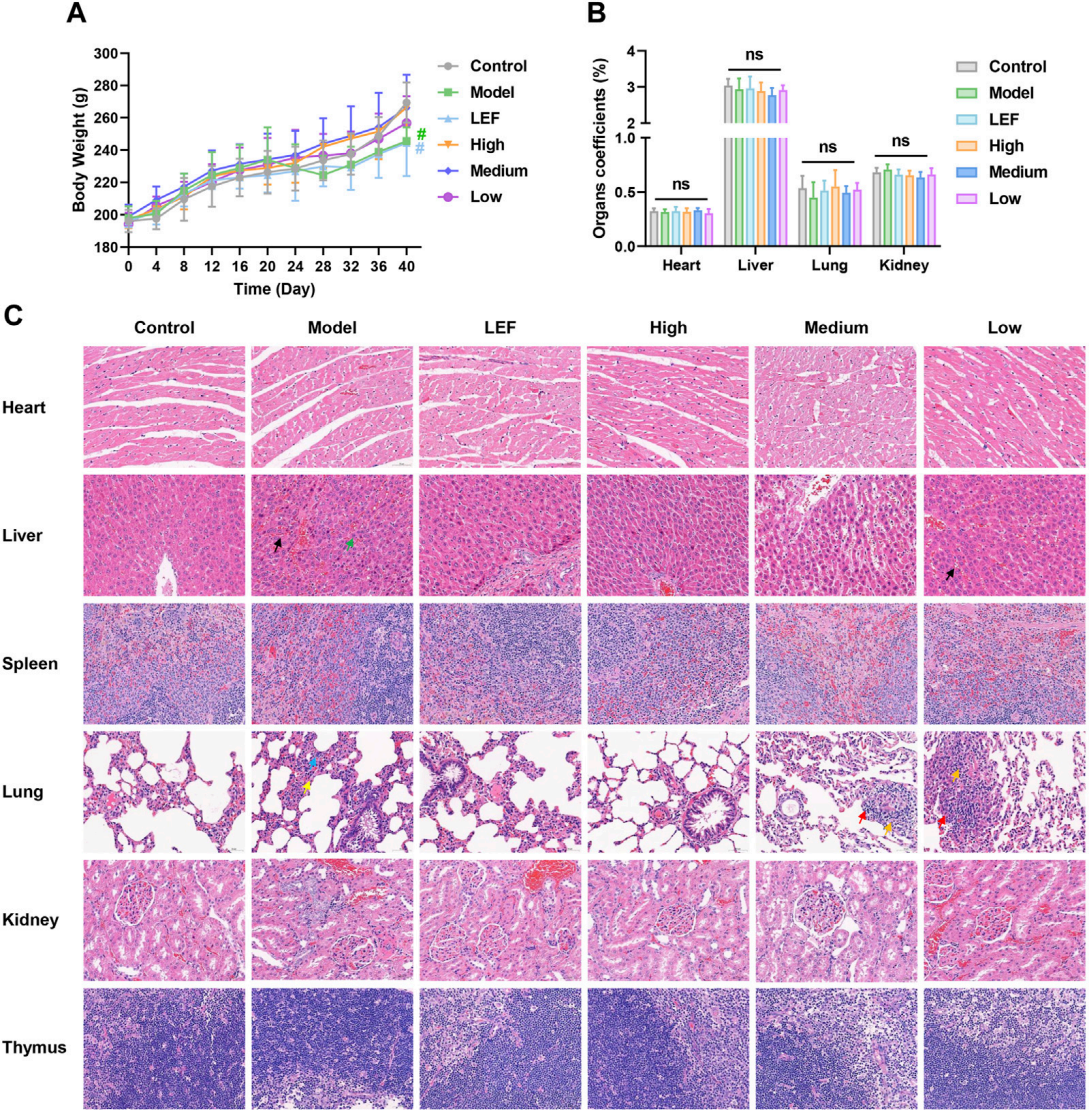
Preliminary evaluation of the treatment safety (Dosages, High group = 9.2, Medium group = 4.6, Low group = 2.3, g/kg/day). **(A)** Body weight change, n = 10. **(B)** Organ coefficients, n = 10. The results are expressed as mean ± SD, *P < 0.05, **P < 0.01, ^ns^ *P* >0.05, compared with the control. **(C)** Histopathological examination of major organs after treatment (×400; n = 6; black arrow: Hepatic sinusoidal congestion; green arrow: hepatic steatosis; blue arrow: proliferation of alveolar epithelial cells; yellow arrow: neutrophils; red arrow: lymphocytes; orange arrow: fibroblast).

Rats in the model group exhibited notable swelling in their hind paws, which subsequently affected their food intake and led to a gradual increase in weight (Figure 5A). The administration of FZD resulted in the alleviation of RA symptoms, leading to an increase in the body weight of rats. As depicted in Figure 5A, high-dose FZD treatment resulted in nearly complete recovery of body weight, comparable to the control group. The body weight serves as evidence of the bio-compatibility of FZD, a confirmation that is further supported by the organ coefficients shown in Figure 5B. Compared to the rats in control groups, no statistical significance was presented, indicating no injury of FZD towards the major organs. The HE results is another convincing evidence, in the Figure 5C. Overall, the HE staining results showed that there were no significant abnormal pathological manifestations in the heart, liver, spleen, lung, kidney, or thymus of the rats, although there were occasional inflammatory cells in individual fields of vision.

### 3.6 FZD exerted potential in modulating key molecular markers associated with RA pathology

**FIGURE 6:**
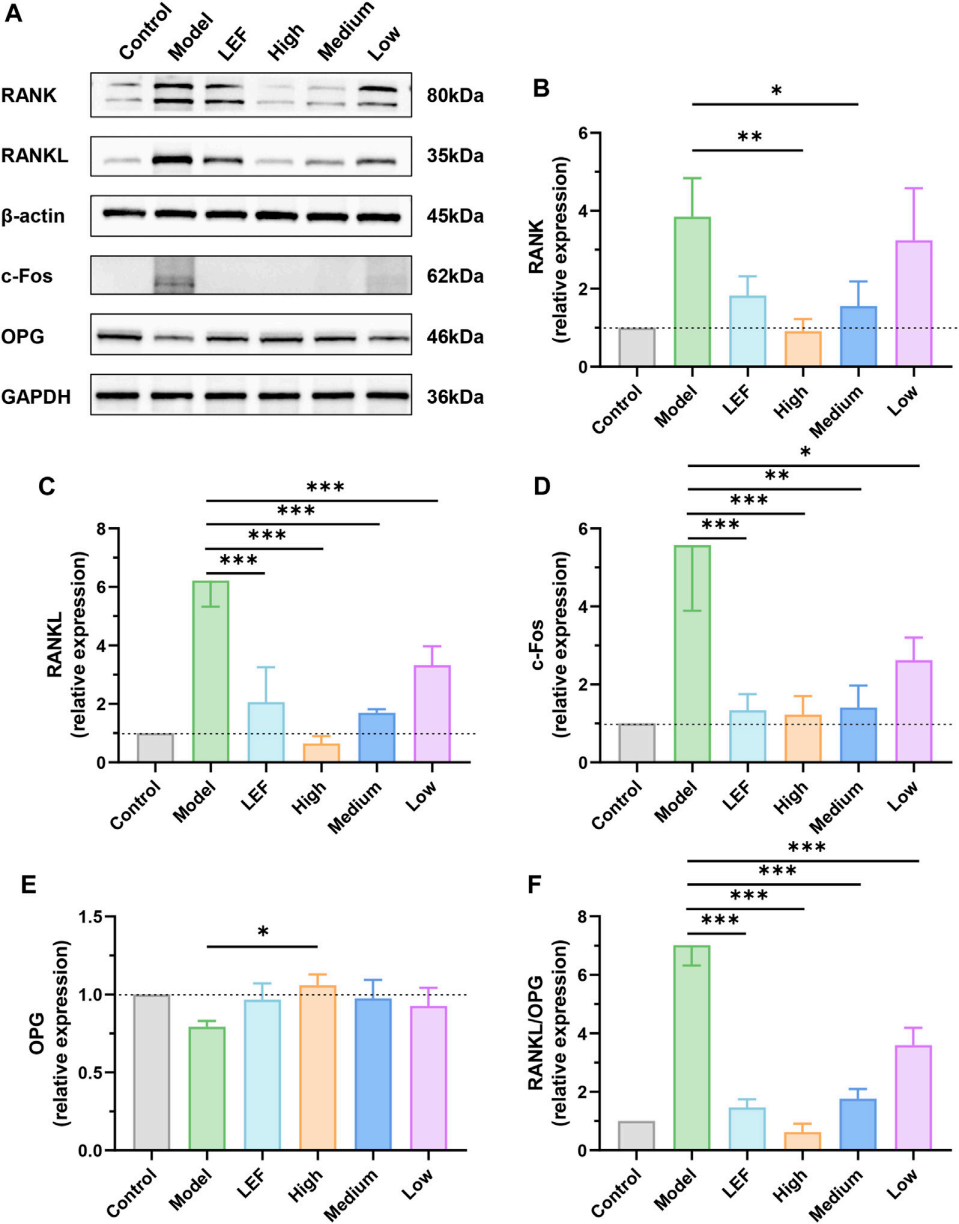
Effect of FZD on the levels of RANK/RANKL signaling pathways-related proteins (Dosages, High group = 9.2, Medium group = 4.6, Low group = 2.3, g/kg/day). **(A)** The protein expression of RANK, RANKL, c-Fos and OPG in the knee joints synovium. The relative expression levels of RANK **(B)**, RANKL **(C)**, c-Fos **(D)** and OPG **(E)**. **(F)** The quantitative analysis of the RANKL/OPG ratio. The protein levels were determined by Western blotting analysis, and the relative protein level was calculated using ImageJ software. The data are shown as the mean ± SD, n = 3, *P < 0.05, **P < 0.01, ***P < 0.001, compared with the model.

In the comprehensive biological study focusing on rat keen joint synovium, Western blotting analysis provided critical insights into the underlying molecular mechanisms (Figure 6). In Figures 6A, B, the model group exhibited a significant increase in RANK levels, indicative of the severity of the observed pathology. Notably, both Leflunomide and FZD exposure led to a downregulation of RANK. The high dose of FZD demonstrated the most pronounced decrease, while the medium exhibited statistically significant reductions compared to the model group. Figure 6A, C focused on RANKL, the ligand for RANK. The model group showed elevated RANKL levels, reflecting an intense inflammatory environment. Treatments, including Leflunomide and FZD exposure, resulted in decreased RANKL levels. The high dose of FZD displayed the most significant reduction, followed by the medium and low doses. Moreover, the proto-oncogene transcription factor (c-Fos), implicated in the progression of RA, was investigated in Figure 6D. The model group demonstrated a substantial increase in c-Fos, indicative of RA severity. Conversely, FZD exposure led to a dose-dependent decrease in c-Fos, suggesting a potential therapeutic effect on inflammatory responses. Contrary to markers indicating pathology, osteoprotegerin (OPG), a beneficial marker for RA, decreased in the model group (Figure 6E). However, Leflunomide and FZD at varying doses, resulted in increased OPG levels, indicating a potential positive impact on RA management. Furthermore, a more precise parameter, the RANKL/OPG ratio, was examined in rats undergoing treatments (Figure 6F). The ratio consistently demonstrated a decrease with FZD exposure. The high dose of FZD exhibited the most significant decrease in the ratio, followed by the Leflunomide-treated, medium dose, and low dose of FZD.

### 3.7 FZD modulated OC performance via the regulation of RANK/RANKL

**FIGURE 7:**
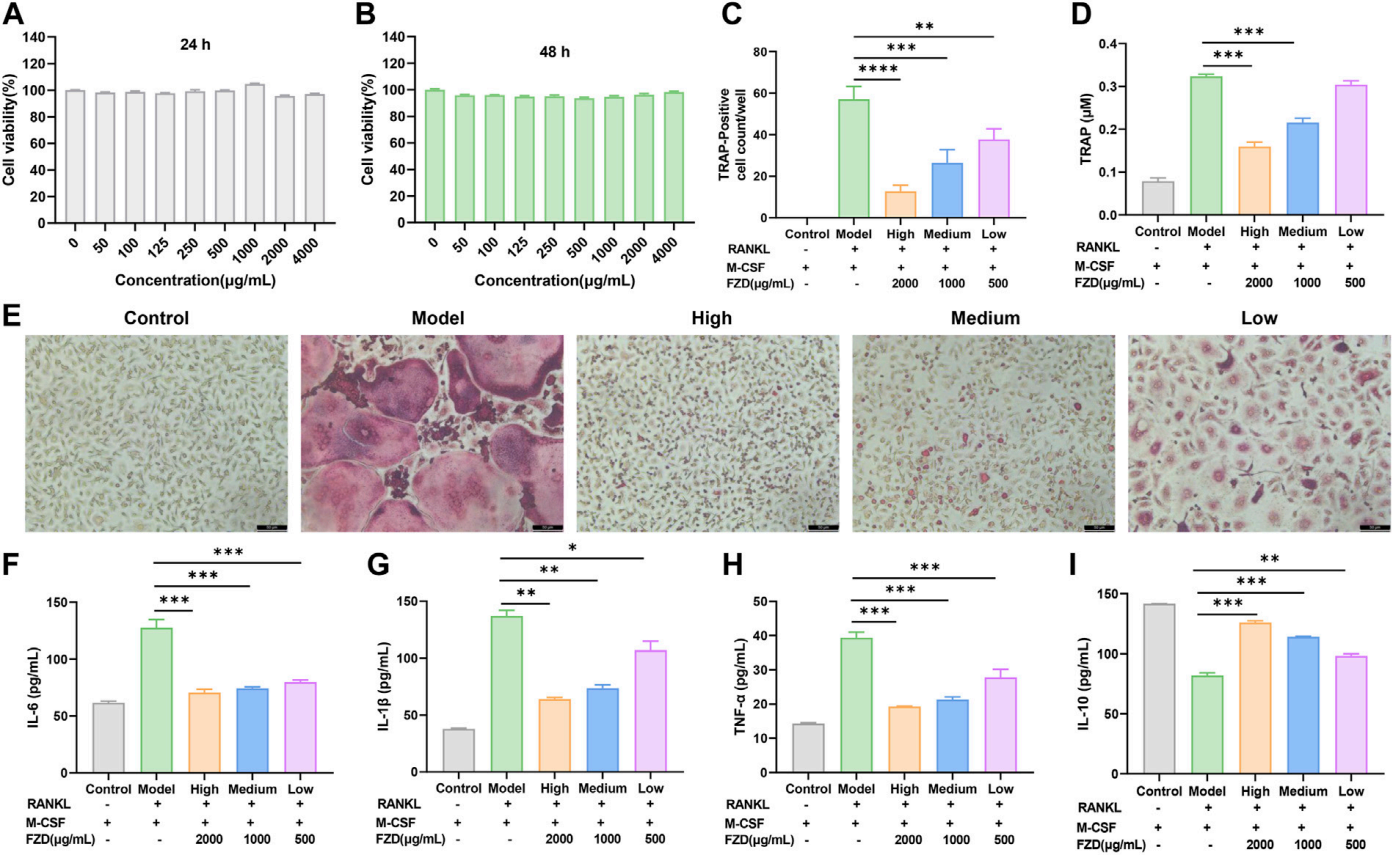
FZD inhibited the differentiation of osteoclasts *in vitro*. **(A)** Cell viability was evaluated by MTT assay (24 h). **(B)** Cell viability was evaluated by MTT assay (48 h). **(C)** The number of TRAP-positive multinucleate osteoclasts. **(D)** TRAP activity in the indicated groups. **(E)** Representative images of TRAP-positive cells in BMMs treated with the indicated concentrations of FZD followed by stimulation with RANKL. Effects of FZD on the levels of IL-6 **(F)**, IL-1β **(G)**, TNF-α **(H)** and IL-10 **(I)** in cellular supernatant. The data are shown as the mean ± SD, n = 3, *P < 0.05, **P < 0.01, ***P < 0.001, ****P < 0.0001, compared with the model.

We evaluated the bio-compatibility of FZD, as shown in Figure 7A, B, the FZD exerted no effects in inhibiting the cell viability. In subsequent experiments investigating the inhibition of osteoclast differentiation and its mechanism, the concentration of FZD was set at 500, 1000, and 2000 μg/mL, representing the low, medium, and high dose groups of FZD, respectively. *In vitro* mechanistic investigation focused on BMMs undergoing differentiation with M-CSF and RANKL. Within the model group, BMMs underwent significant transformation into osteoclasts, as indicated by TRAP staining, with approximately 60 giant multinuclear cells per well (Figure 7C). However, in the presence of FZD at varying doses, the number of TRAP-positive cells dramatically decreased. The high dose FZD group exhibited a remarkable three-fold reduction in osteoclasts. Even with low-dose FZD treatment, the conversion of BMMs to osteoclasts was significantly lower than in the model group, demonstrating statistical significance. Moreover, a quantitative analysis of TRAP activity was conducted using a TRAP kit. The group induced by RANKL and M-CSF showed the highest level of TRAP, followed by the addition of FZD at low, medium, and high doses (Figure 7D). Figure 7F shows that RANKL and M-CSF increased IL-6 levels, which were reduced by FZD. Similarly, FZD inhibited elevated IL-1β (Figure 7G), with the high dose being most effective. In Figure 7H, TNF-α levels decreased with FZD treatment compared to the model group, indicating anti-inflammatory potential. FZD also elevated IL-10 levels, with the high dose showing the most significant increase (Figure 7I). To determine the specific impact of FZD treatment on BMMs, the effects of FZD treatment on BMMs were assessed independently, and the results indicated that varying concentrations of FZD did not exert a significant impact on the differentiation of BMMs into osteoclasts alone (Supplementary Figure S1).

### 3.8 FZD modulated key molecular players involved in bone metabolism and inflammatory responses

**FIGURE 8:**
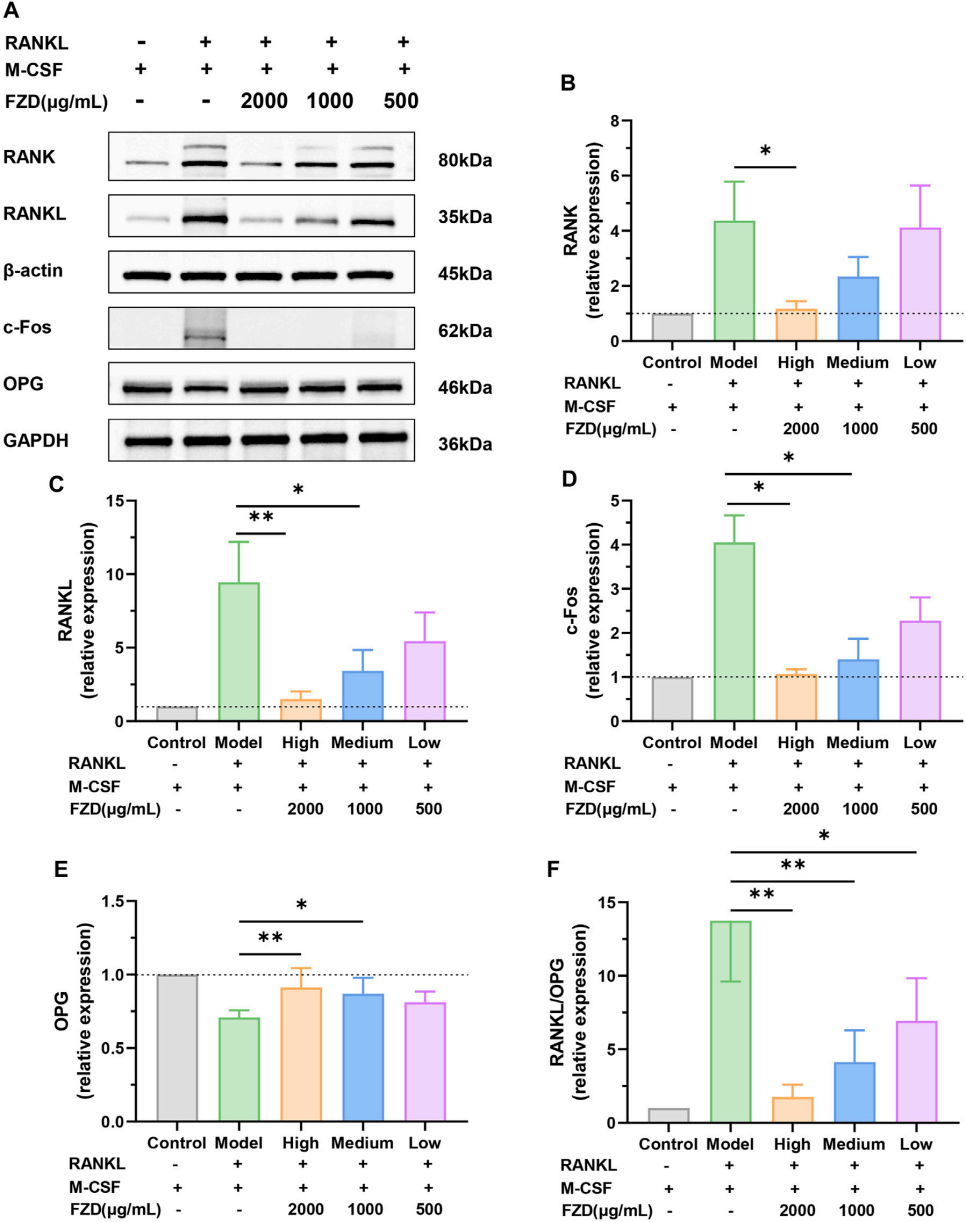
FZD suppressed the RANK/RANKL signaling pathway. **(A)** Representative immunoblots of RANK, RANKL, c-Fos and OPG in BMMs were performed by Western blotting. The relative expression levels of RANK **(B)**, RANKL **(C)**, c-Fos **(D)** and OPG **(E)** in the indicated groups. **(F)** The quantitative analysis of the RANKL/OPG ratio. The protein levels were determined by Western blotting analysis, and the relative protein level was calculated using ImageJ software. The data are shown as the mean ± SD, n = 3, *P< 0.05, **P< 0.01, ***P< 0.001, compared with the model.

In addition to assessing soluble inflammation-associated factors, our investigation also examined the effect of FZD on the RANK/RANKL signaling pathway through Western blotting. Figure 8A, B vividly illustrates that the induction of RANKL plus M-CSF resulted in an elevation of RANK levels. Intriguingly, the addition of FZD exhibited a remarkable downregulatory effect on RANK. High doses of FZD resulted in a marked reduction in RANK levels, while medium and low doses exhibited less dramatic reductions compared to the high dose. Furthermore, the levels of RANKL also displayed a decreasing trend (Figure 8C). The initial high level of RANK induced by the RANKL and M-CSF was effectively mitigated by the addition of FZD. The high dose of FZD, in particular, exerted the most potent downregulatory effects. Figure 8D shows that RANKL and M-CSF induction increased c-Fos levels, whereas FZD treatment led to a decrease in c-Fos expression. Contrastingly, OPG, an opposing marker, displayed increased levels upon FZD treatment compared to the model group, suggesting a potential regulatory effect of FZD on bone homeostasis (Figure 8E). The RANKL/OPG ratio provides compelling evidence, as the model group exhibited the highest value, while exposure to FZD resulted in a significant decrease (Figure 8F). This observation supports the notion that FZD, particularly at higher concentrations, can effectively modulate the RANKL/OPG ratio, indicating a potential therapeutic impact on RA.

## 4 Discussion

RA can cause irreversible destruction of joints and resulted in disability of patients (de Punder and van Riel, 2011). In the process of RA, effector cells and immune cells, such as fibroblast-like synoviocytes (RAFLS), helper T cell 17 (Th17), and M1-type macrophages, release inflammatory cytokines including TNF-α, IL-1β, IL-6, and IL-17, which then lead to the secretion of RANKL (Danks et al., 2016; Muñoz et al., 2020; Nygaard and Firestein, 2020; Chen et al., 2021). The upregulation RANKL promotes the differentiation of osteoclast precursor cells into mature osteoclasts. The excessive proliferation and activation of these osteoclasts disrupt the delicate equilibrium between osteoclasts and osteoblasts, ultimately leading to an imbalance in the bone microenvironment, resulting in bone destruction and structural disruption (Komatsu and Takayanagi, 2022).

FZD is a traditional Chinese formula and the application of FZD on RA remains significant. To investigate the impact of FZD in RA, we established collagen-induced arthritis (CIA) model. CIA is extensively studied as an animal model due to its similar pathological and immunological features with human RA (Kannan et al., 2005). CIA models illustrate that autoimmunity directed towards collagen type II (CII) can result in autoimmune arthritis, characterized by bone erosion, cartilage destruction, and inflammation of synovial joints (Cho et al., 2007; Asquith et al., 2009). Consequently, animal models like CIA mimic human RA closely and has been considered the gold standard *in vivo* model for RA studies (Asquith et al., 2009; Poutoglidou et al., 2020). Taking into consideration the higher incidence of RA in females compared to males (Finckh et al., 2022), and based on previous research findings (Wang K. et al., 2018; Zhao et al., 2021), we utilized a female CIA rat model as an *in vivo* model for this study, despite potential hormonal influences on immune response in female rats. In our therapeutic assessment, FZD significantly inhibit the ankle swelling in CIA model and reduce the volume of rat toe. Furthermore, FZD decrease the paw thickness, which dramatically increase in the model group. In addition, the reduction in the spleen index in the FZD treated group also confirmed the potential of FZD on RA regulation. The body weight presents no obvious change over time in treatment group, indicating the excellent biosafety of FZD. No impairments were found in the organs of rats exposed to FZD, as indicated by organ coefficients and HE staining results.

In the mechanistic study, inspired by other investigations, the interplay between inflammation and osteoclast activity is central to the development and progression of RA, suggesting the importance of effectively managing both aspects as integral components of treatment (Maeda et al., 2022; Niu et al., 2022). Massive OC in RA impede the natural repair process and prevent the cure of RA (Baum and Gravallese, 2016). Osteoclast maturation involves several stages, including preosteoclasts, non-functional polykaryons, and mature osteoclasts (Kim et al., 2020). The ligand of receptor activator of nuclear factor κB ligand (RANKL) is a major factor for these processes (Sun et al., 2021). Increased RANKL expression and/or decreased OPG expression were classic features in RA (Skoumal et al., 2005). The alteration of FZD in the ratio of RANKL/OPG in our study suggested the possible target of FZD is RANKL and OPG. Both of which were confirmed with *in vitro* and *in vivo* study and also consistent with other’s findings (Chiba et al., 2009; Quaresma et al., 2023). As stated, the RANKL serves as crucial paracrine system ligand, participating in osteoclast formation and differentiation (Honma et al., 2021). And FZD inhibit the RANKL levels. With the associated logic chain, receptor activator of nuclear factor κB (RANK) interact with RANKL, thus RANK also play critical roles in promoting the formation of mature osteoclast cells (Hofbauer and Heufelder, 2001; Li B. et al., 2022). On the contrary, the OPG, a soluble decoy receptor for RANKL, competitively binds with RANKL, thereby blocking the interaction between RANKL and RANK and benefiting RA treatment (Udagawa et al., 2021). In our study, FZD increase the OPG, which act as a brake in the bone microenvironment, preventing excessive osteoclasts and the resultant abnormal bone destruction. In summary, FZD decreased RANKL levels, increased OPG levels, and reduced the RANKL/OPG ratio, inhibiting osteoclast activity and alleviating bone destruction.

The abnormal ratio of RANKL/OPG is closely associated with inflammatory cytokines in RA. Key cytokines such as IL-1β have been implicated in increasing bone resorption and fracture rates by elevating RANKL levels (Wang Y. et al., 2018). Additionally, TNF-α and IL-6 also contribute to bone loss (Marahleh et al., 2019; Takeuchi et al., 2021). Our study indicates that FZD enhances the inhibition of the inflammatory factors including IL-6 and TNF-α, confirming the potential of FZD on bone activity. Conversely, anti-inflammatory cytokines, which can inhibit osteoclast-genesis, are found to be decreased in RA (Chen et al., 2019).

Classically, BMMs were exposed to RANKL and M-CSF to induce osteoclast differentiation (Jacome-Galarza et al., 2019), upon binding to the highly expressed RANK on the surface of preosteoclast cells, RANKL triggers their differentiation into mature osteoclasts with the induction of M-CSF. The impact of FZD on BMMs treated only with RANKL, in the absence of M-CSF, remains unknown. Therefore, we investigated M-CSF’s role in BMMs and confirmed that M-CSF alone does not induce osteoclast differentiation. We then used both M-CSF and RANKL to induce BMMs differentiation into osteoclasts to further understand the combined effect. The data demonstrates that FZD significantly inhibits RANKL-induced osteoclast differentiation by reducing the number of osteoclasts in a dose-dependent manner.

Apart from impacting the critical receptors RANK and OPG in the RANK/RANKL signaling pathway, FZD may also exert its effects by influencing other receptors. In the HPLC fingerprints, eight compounds were identified as follows: gallic acid, albiflorin, paeoniflorin, 1,2,3,4,6-O-pentagalloylglucose, benzoylmesaconine, benzoyl aconitine, benzoylpaeoniflorin, and atractylenolide III. And some of these compounds have been found to influence osteoclast differentiation. Nuclear factor of activated T cells 1 (NFATc1) is a pivotal transcription factor that plays a critical role in driving osteoclastogenesis, which encompasses the processes of both osteoclast formation and maturation (Lorenzo, 2017). A study has shown that gallic acid inhibits the upregulation of NFATc1 expression induced by RANKL, as well as the activation of the p38 signaling pathway. Additionally, it prevents the RANKL-mediated downregulation of interferon regulatory factor-8 (IRF-8), which recognized as an anti-osteoclastogenic factor that suppresses NFATc1 expression (Shim et al., 2015). In studies related to paeoniflorin, ginsenoside Rg2 and poria cocos polysaccharide (PCP), they have demonstrated similar inhibitory effects on NFATc1 (Li et al., 2018; Song et al., 2018; Lee et al., 2023). Ginsenoside Rh2 is also a significant essentials in FZD efficacy in inhibiting RANKL-induced OC, specifically, ginsenoside Rh2 affect the expression of transcription factors, c-Fos and NFATc1, as well as osteoclast markers, TRAP and OSCAR (He et al., 2012). Additionally, paeoniflorin effectively ameliorates collagen-induced arthritis by inhibiting the NF-κB signaling pathway in osteoclast differentiation. The research findings show that paeoniflorin downregulates the expression of genes associated with bone destruction, such as TRAP, MMP-9, MMP-3, and RANKL, and interferes with osteoclast differentiation by suppressing p65 phosphorylation (Xu et al., 2018). The ERK, JNK, and p38 MAPK pathways are crucial for transmitting signals from RANK to the nucleus, leading to the activation of transcription factors like c-Fos (Jiang et al., 2023). Afterwards, c-Fos impacts the RANK/RANKL by regulating RANKL expression (Tong et al., 2021). In our study, FZD have impact on the levels of c-Fos both *in vivo* and *in vitro*. In addition, The FZD roles may associate with the regulation of ginsenoside Rg2 on the phosphorylation of mitogen-activated protein kinases (Ma et al., 2024). Hence, NFATc1 and receptors on the NF-κB, ERK, JNK, and p38 MAPK signaling pathways could serve as potential targets for further investigation regarding the impact of FZD.

## 5 Conclusion

**FIGURE 9:**
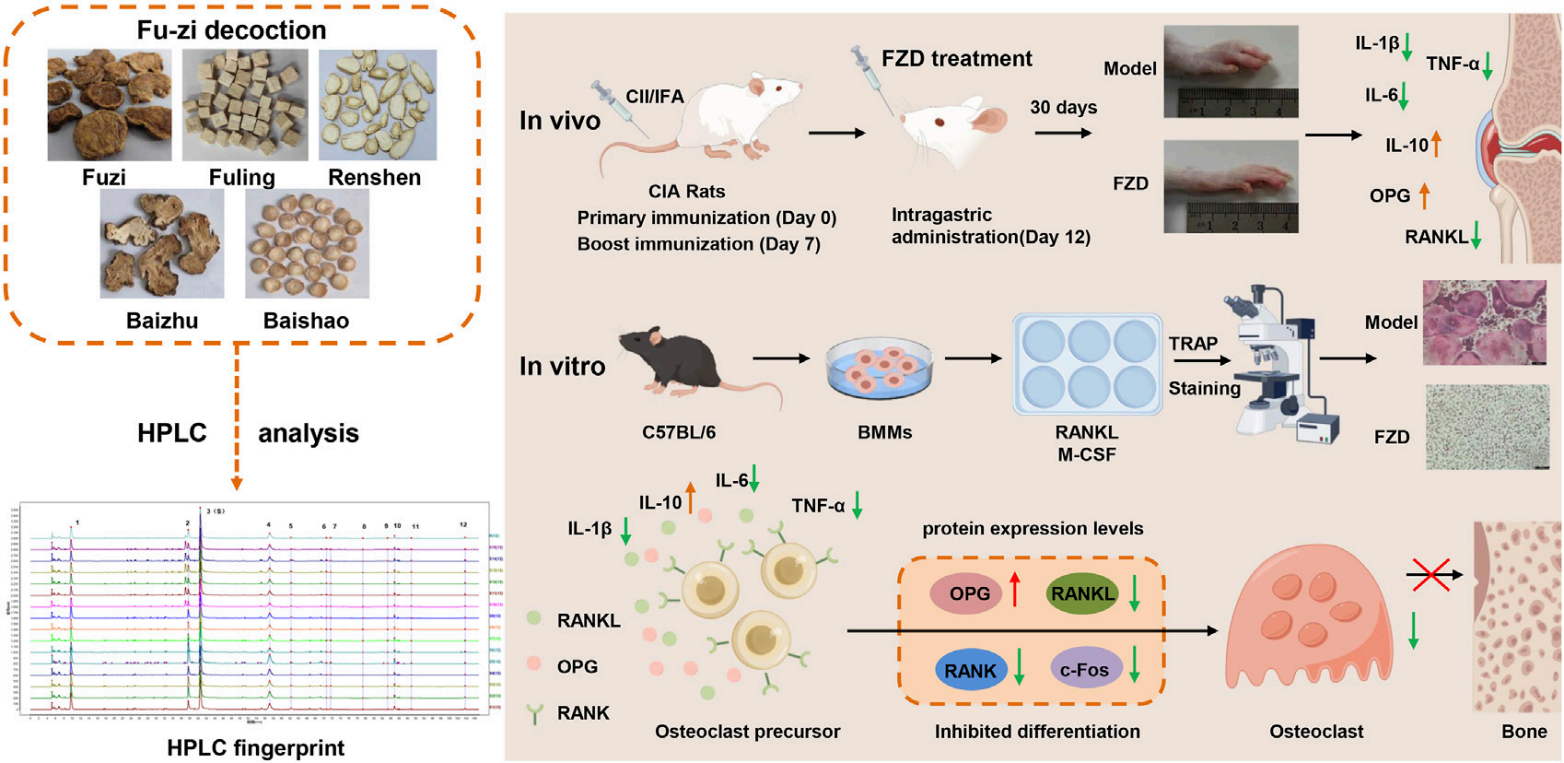
A schematic diagram illustrating Fu-zi decoction in attenuating rheumatoid arthritis by modulating RANK/RANKL signaling pathway.

As summarized in Figure 9, the present study showed that FZD could suppress the onset and progression of collagen-induced arthritis, as evaluated using arthritis index scoring, foot swelling assessment, histopathological assessment, micro-CT, and the expression of cytokines and related proteins. Although the precise mechanisms behind these effects were not fully elucidated, our findings indicate that FZD has anti-inflammatory and anti-RA properties by modulating the RANK/RANKL signaling pathway and reducing osteoclast differentiation both *in vivo* and *in vitro*. These findings provide a theoretical framework for potential clinical application of FZD in RA treatment.

## Data Availability

The original contributions presented in the study are included in the article/Supplementary Material, further inquiries can be directed to the corresponding author.
