# Supplementary figures and images for: Fu-zi decoction attenuate rheumatoid arthritis in vivo and in vitro by modulating RANK/RANKL signaling pathway
Source: Front Pharmacol. 2024 Jul 23;15:1423884. doi: 10.3389/fphar.2024.1423884 (PMC11300212; doi:10.3389/fphar.2024.1423884)

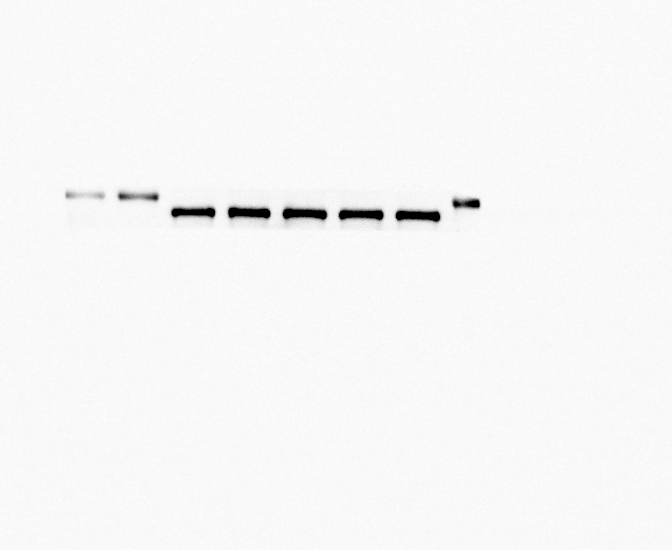

Supplement: Supplementary file 1 [file DataSheet2.zip › origin WB image/1 cell actin _8bit.tif]

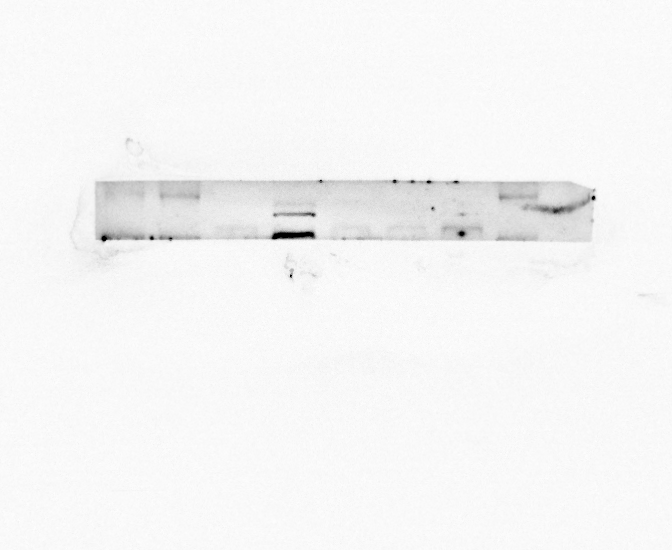

Supplement: Supplementary file 1 [file DataSheet2.zip › origin WB image/1 cell c-Fos_8bit.tif]

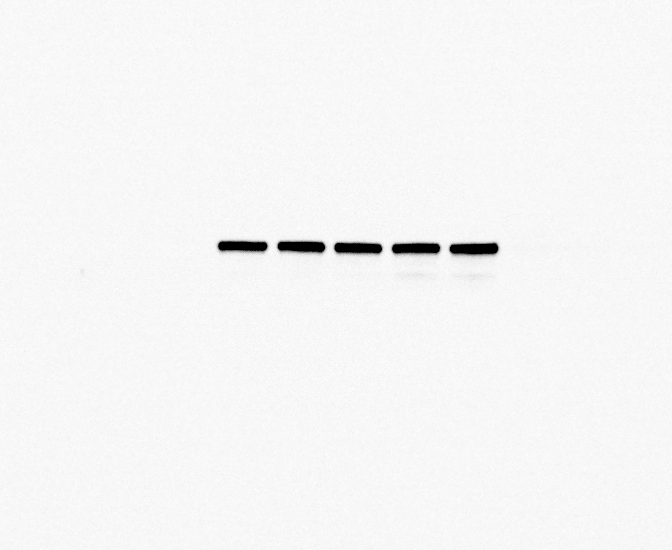

Supplement: Supplementary file 1 [file DataSheet2.zip › origin WB image/1 cell GAPDH_8bit.tif]

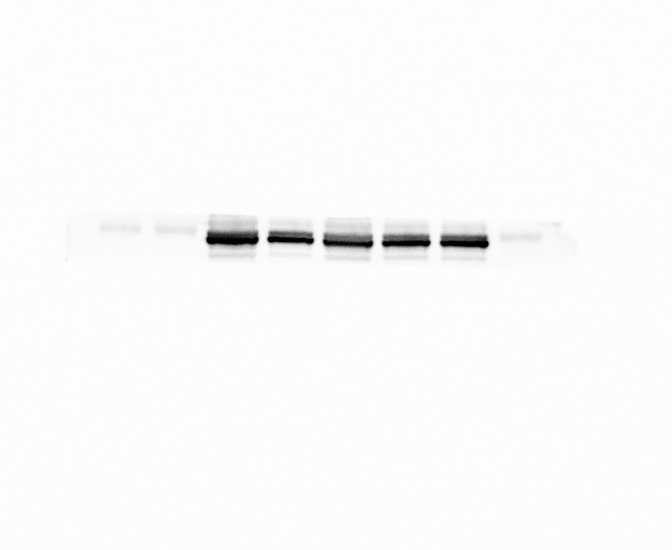

Supplement: Supplementary file 1 [file DataSheet2.zip › origin WB image/1 cell OPG_8bit.tif]

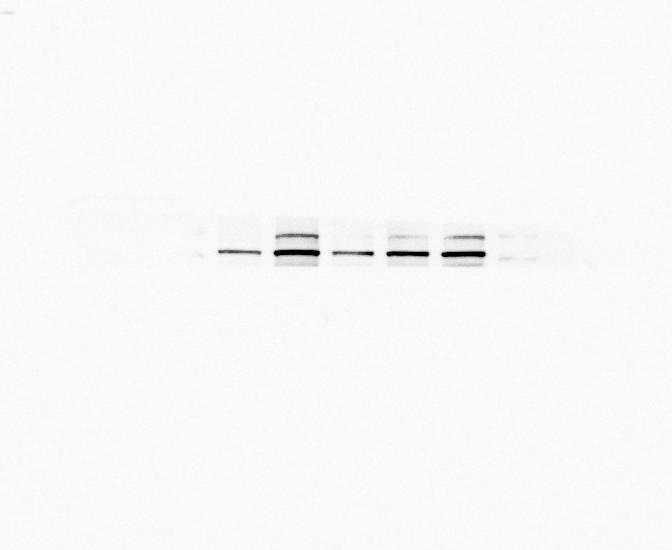

Supplement: Supplementary file 1 [file DataSheet2.zip › origin WB image/1 cell RANK _8bit.tif]

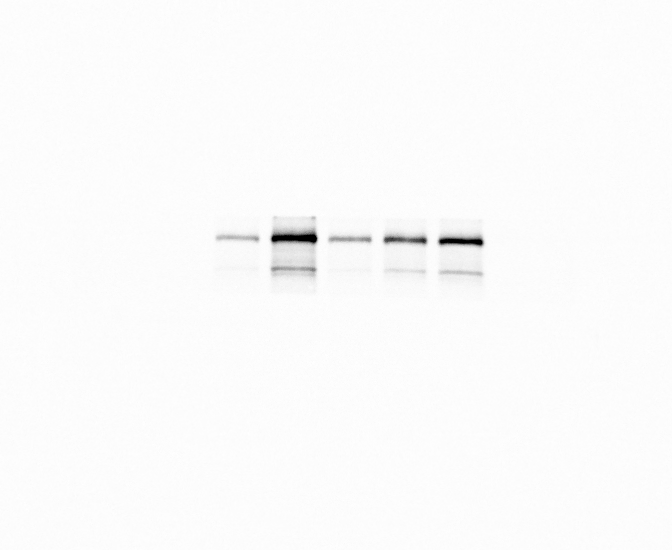

Supplement: Supplementary file 1 [file DataSheet2.zip › origin WB image/1 cell RANKL_8bit.tif]

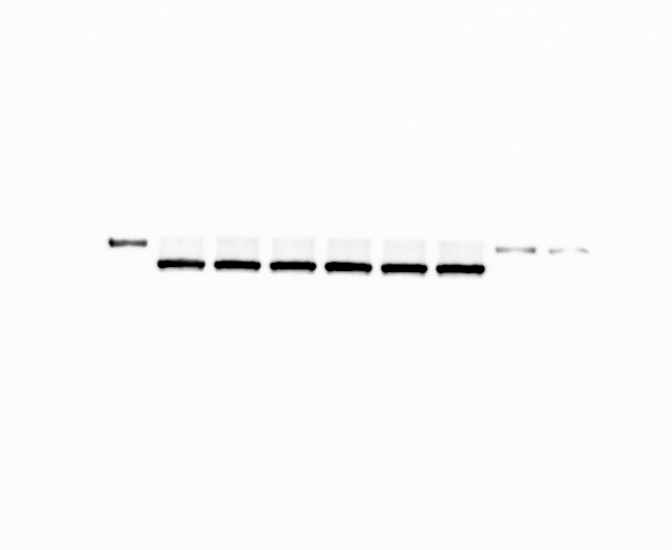

Supplement: Supplementary file 1 [file DataSheet2.zip › origin WB image/1 CIA actin _8bit.tif]

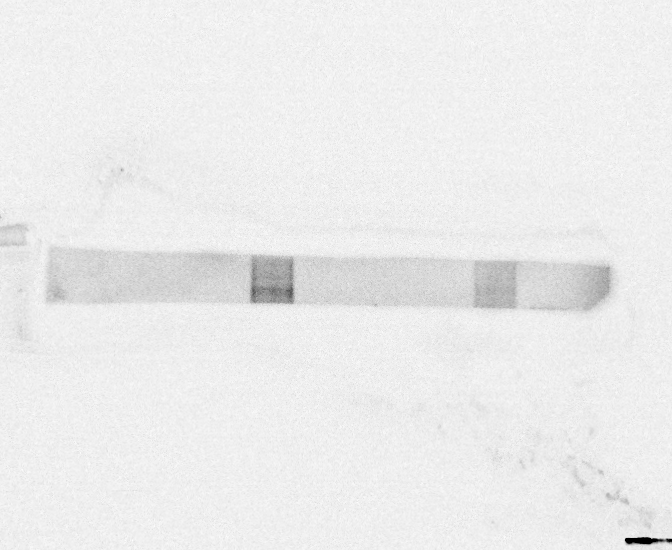

Supplement: Supplementary file 1 [file DataSheet2.zip › origin WB image/1 CIA c-Fos_8bit.tif]

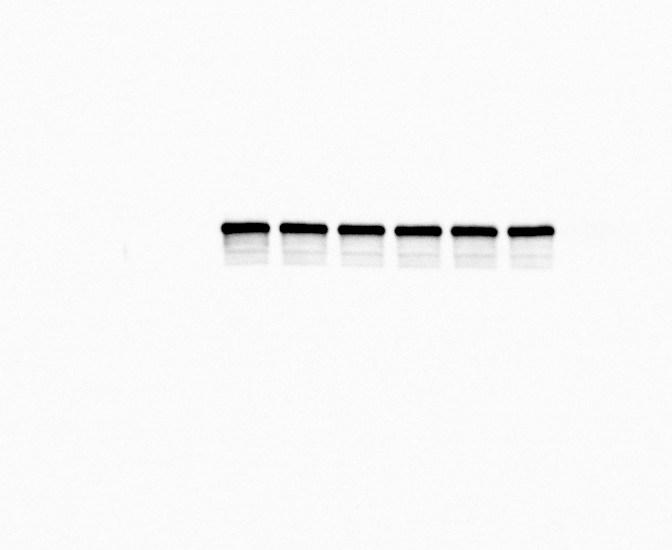

Supplement: Supplementary file 1 [file DataSheet2.zip › origin WB image/1 CIA GAPDH_8bit.tif]

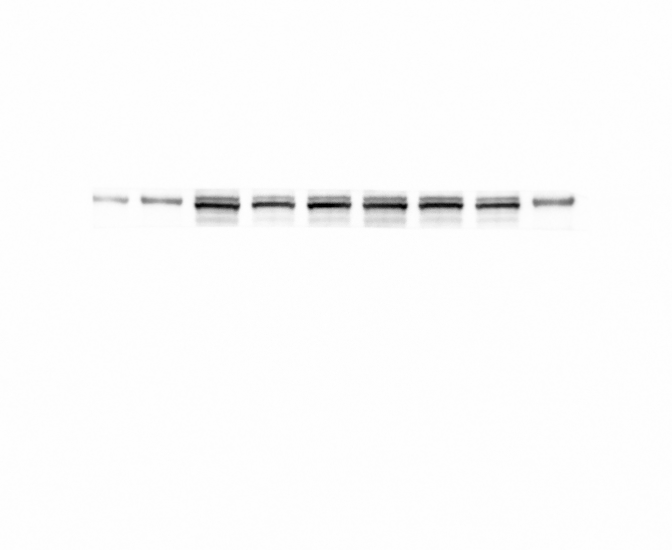

Supplement: Supplementary file 1 [file DataSheet2.zip › origin WB image/1 CIA OPG_8bit.tif]

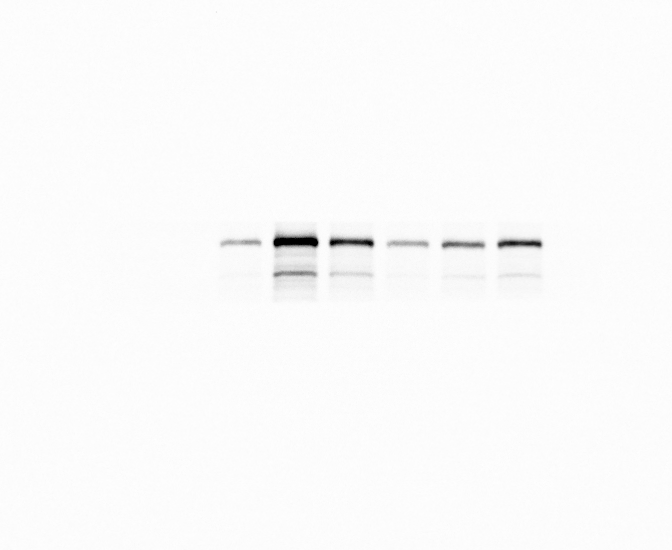

Supplement: Supplementary file 1 [file DataSheet2.zip › origin WB image/1 CIA RANKL_8bit.tif]

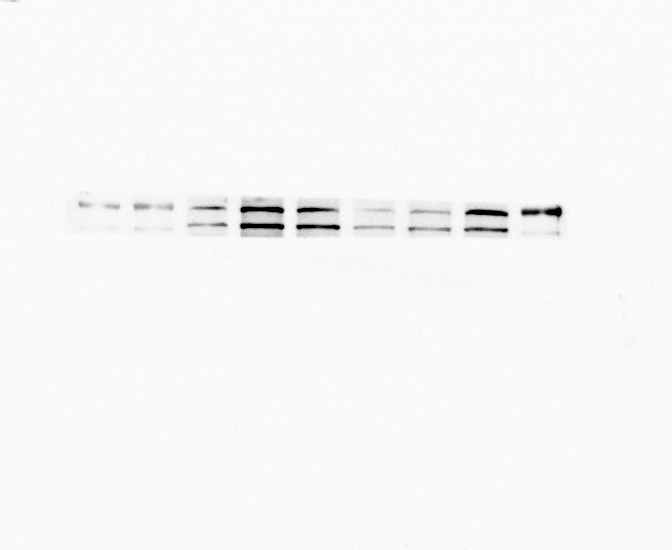

Supplement: Supplementary file 1 [file DataSheet2.zip › origin WB image/1 CIA RANK_8bit.tif]

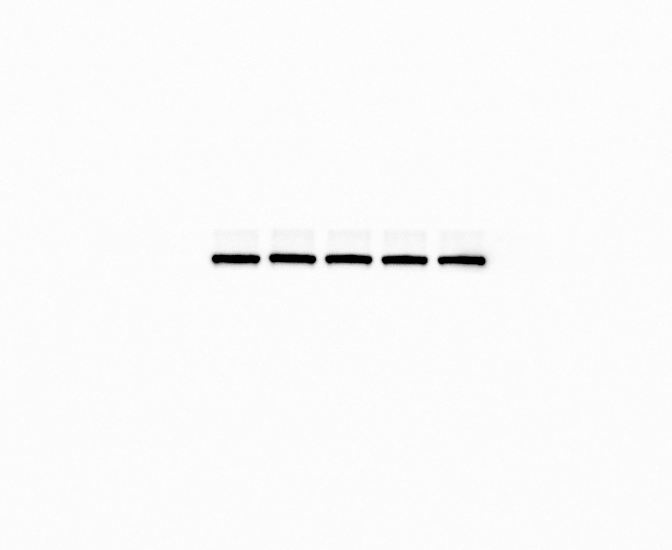

Supplement: Supplementary file 1 [file DataSheet2.zip › origin WB image/2 cell actin_8bit.tif]

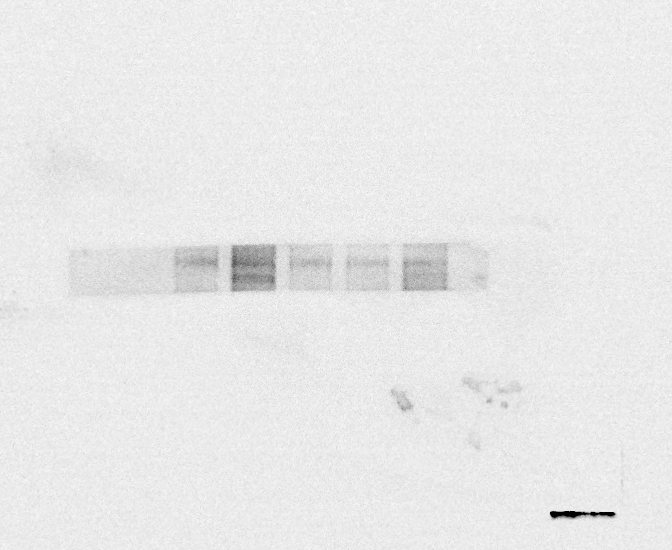

Supplement: Supplementary file 1 [file DataSheet2.zip › origin WB image/2 cell c-Fos_8bit.tif]

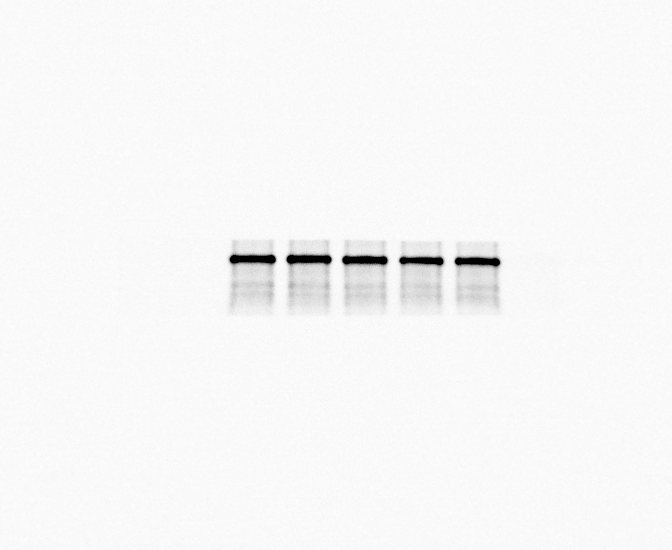

Supplement: Supplementary file 1 [file DataSheet2.zip › origin WB image/2 cell GAPDH_8bit.tif]

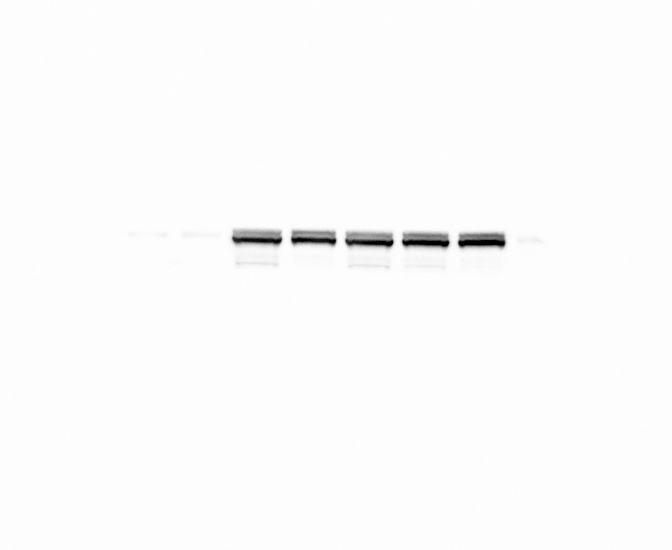

Supplement: Supplementary file 1 [file DataSheet2.zip › origin WB image/2 cell OPG_8bit.tif]

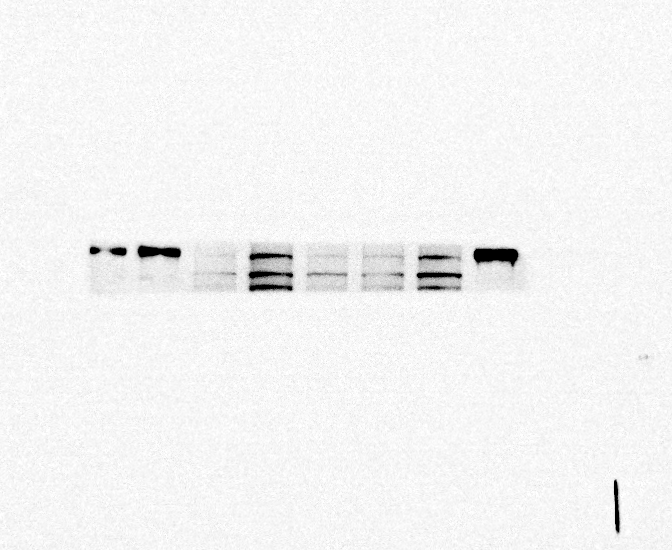

Supplement: Supplementary file 1 [file DataSheet2.zip › origin WB image/2 cell RANK _8bit.tif]

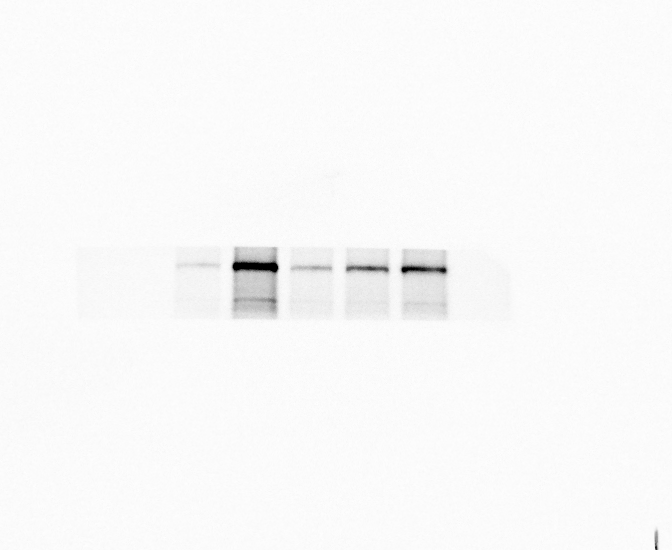

Supplement: Supplementary file 1 [file DataSheet2.zip › origin WB image/2 cell RANKL_8bit.tif]

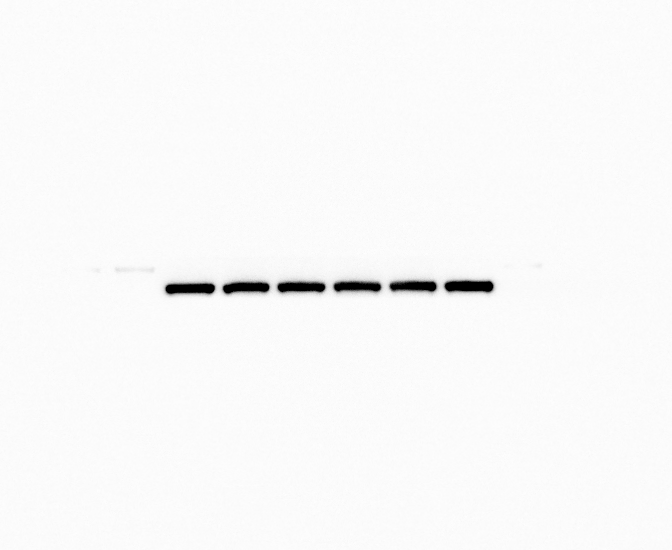

Supplement: Supplementary file 1 [file DataSheet2.zip › origin WB image/2 CIA actin_8bit.tif]

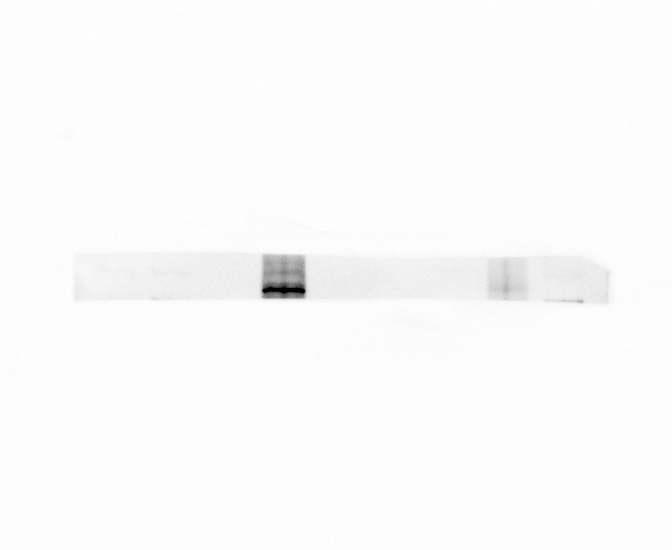

Supplement: Supplementary file 1 [file DataSheet2.zip › origin WB image/2 CIA c-Fos_8bit.tif]

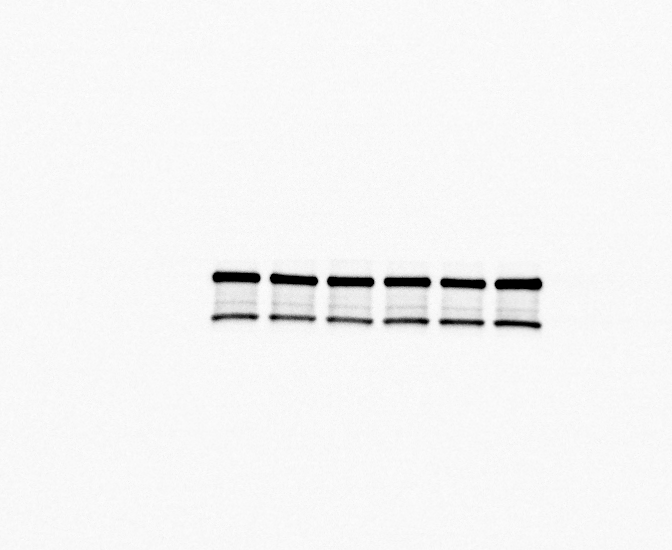

Supplement: Supplementary file 1 [file DataSheet2.zip › origin WB image/2 CIA GAPDH_8bit.tif]

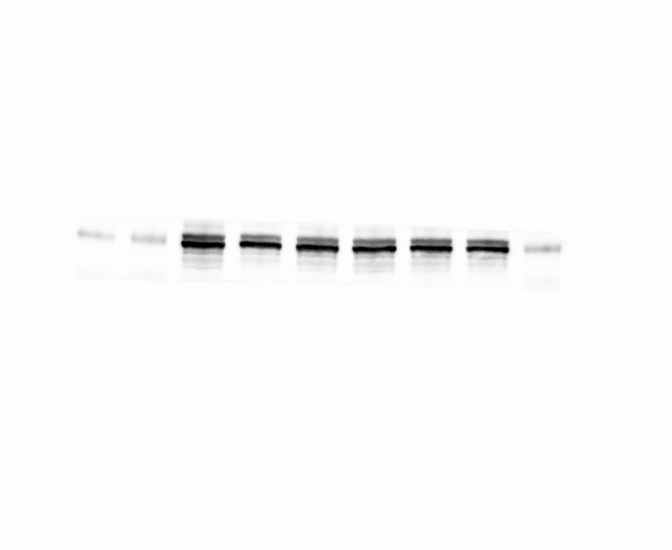

Supplement: Supplementary file 1 [file DataSheet2.zip › origin WB image/2 CIA OPG_8bit.tif]

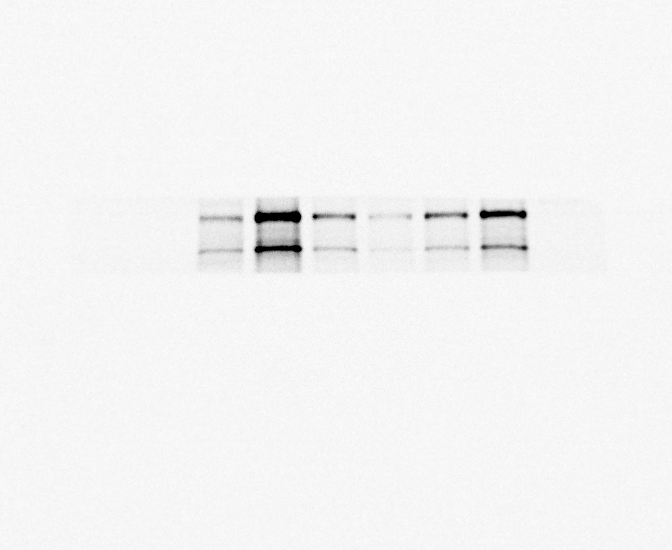

Supplement: Supplementary file 1 [file DataSheet2.zip › origin WB image/2 CIA RANKL_8bit.tif]

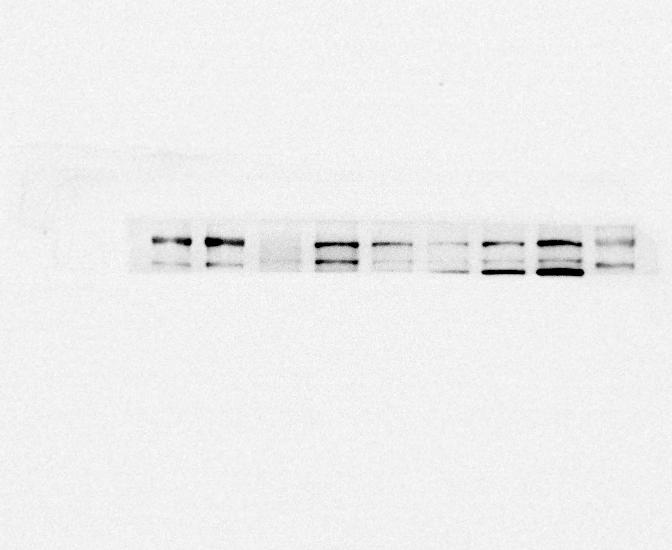

Supplement: Supplementary file 1 [file DataSheet2.zip › origin WB image/2 CIA RANK_8bit.tif]

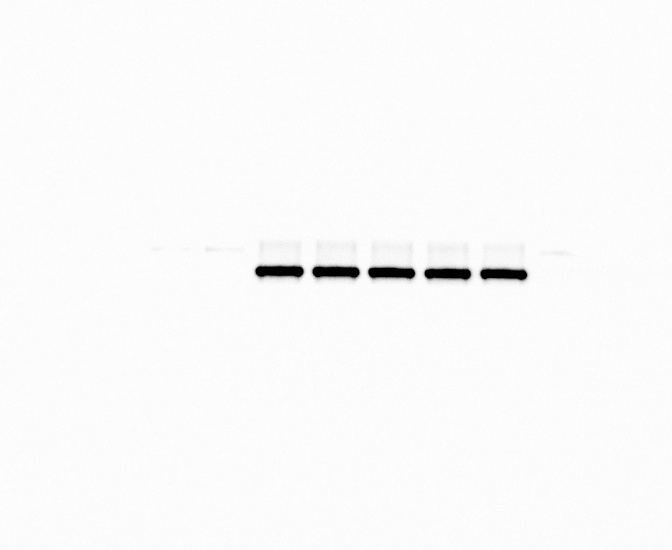

Supplement: Supplementary file 1 [file DataSheet2.zip › origin WB image/3 cell actin_8bit.tif]

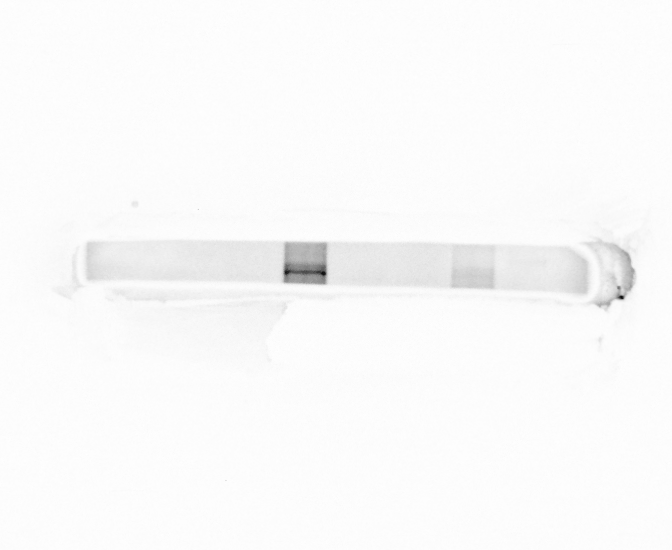

Supplement: Supplementary file 1 [file DataSheet2.zip › origin WB image/3 cell c-Fos _8bit.tif]

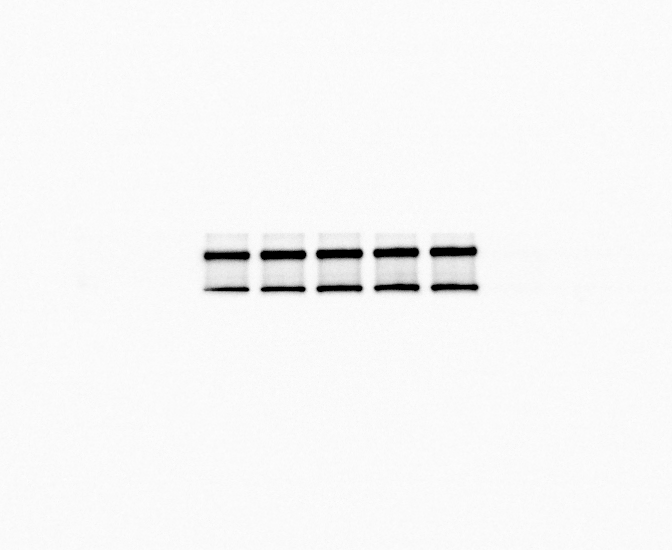

Supplement: Supplementary file 1 [file DataSheet2.zip › origin WB image/3 cell GAPDH_8bit.tif]

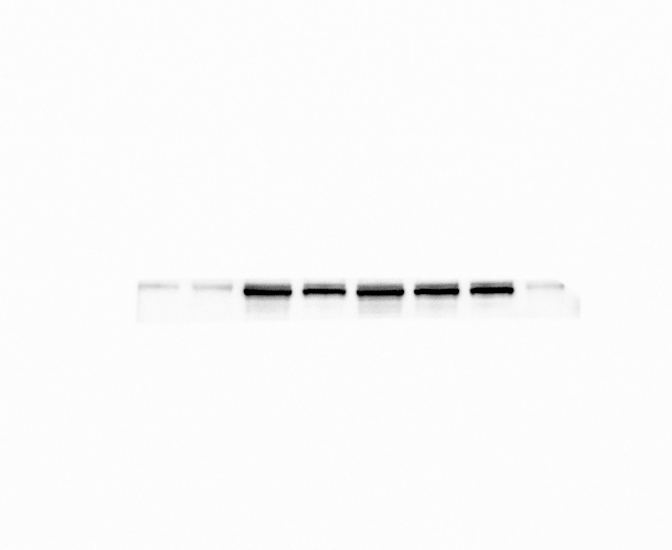

Supplement: Supplementary file 1 [file DataSheet2.zip › origin WB image/3 cell OPG_8bit.tif]

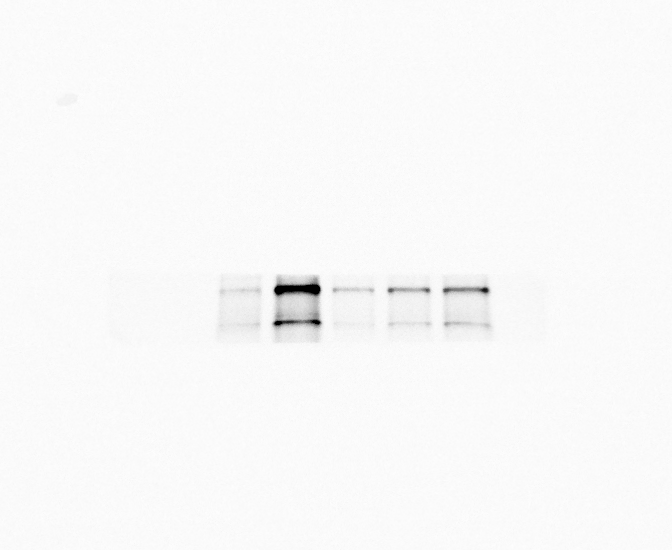

Supplement: Supplementary file 1 [file DataSheet2.zip › origin WB image/3 cell RANKL_8bit.tif]

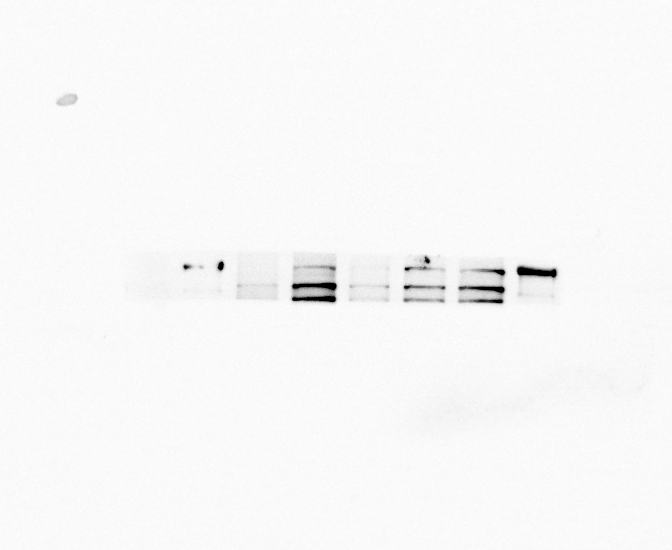

Supplement: Supplementary file 1 [file DataSheet2.zip › origin WB image/3 cell RANK_8bit.tif]

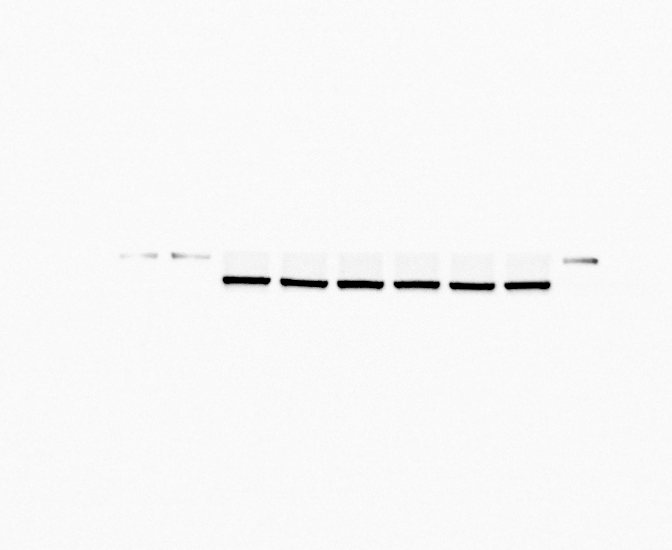

Supplement: Supplementary file 1 [file DataSheet2.zip › origin WB image/3 CIA actin_8bit.tif]

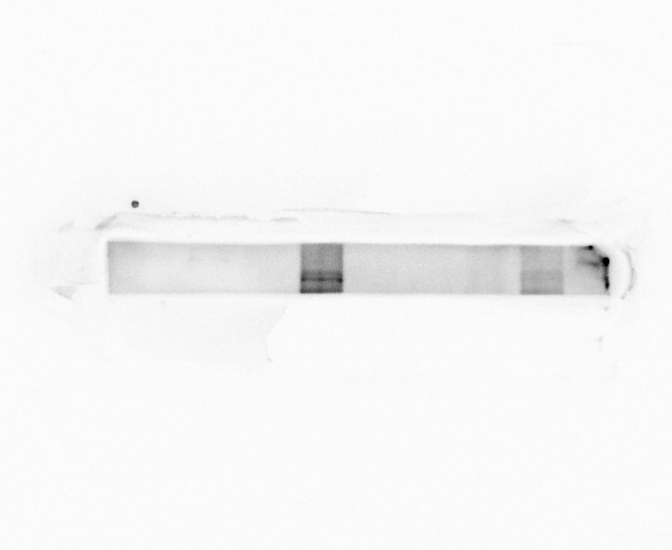

Supplement: Supplementary file 1 [file DataSheet2.zip › origin WB image/3 CIA c-Fos _8bit.tif]

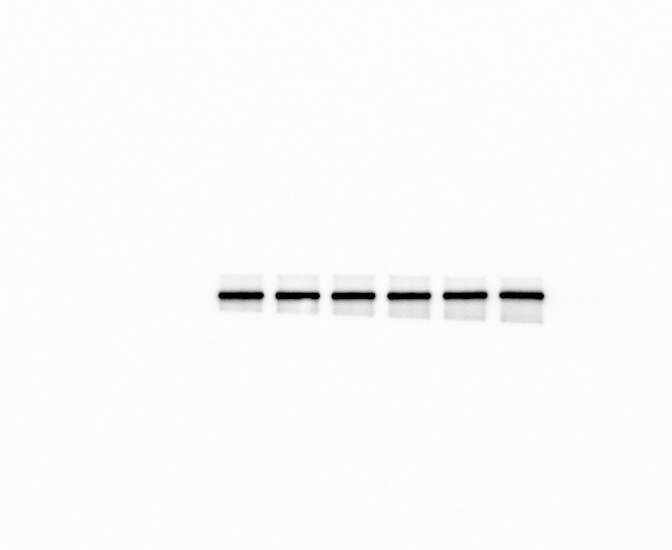

Supplement: Supplementary file 1 [file DataSheet2.zip › origin WB image/3 CIA GAPDH_8bit.tif]

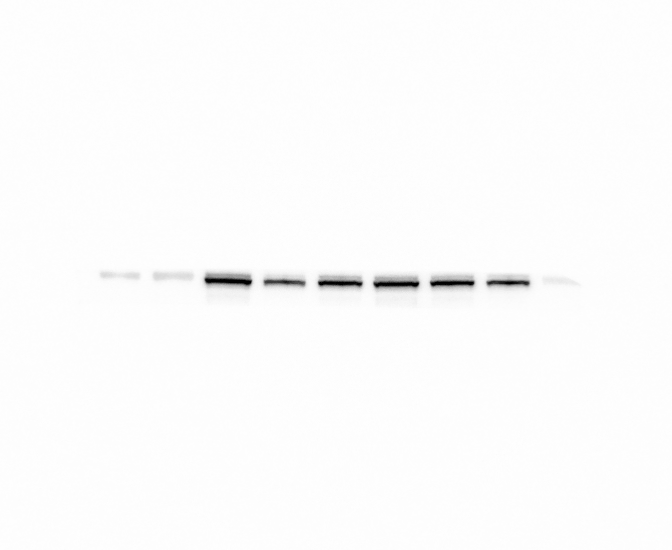

Supplement: Supplementary file 1 [file DataSheet2.zip › origin WB image/3 CIA OPG _8bit.tif]

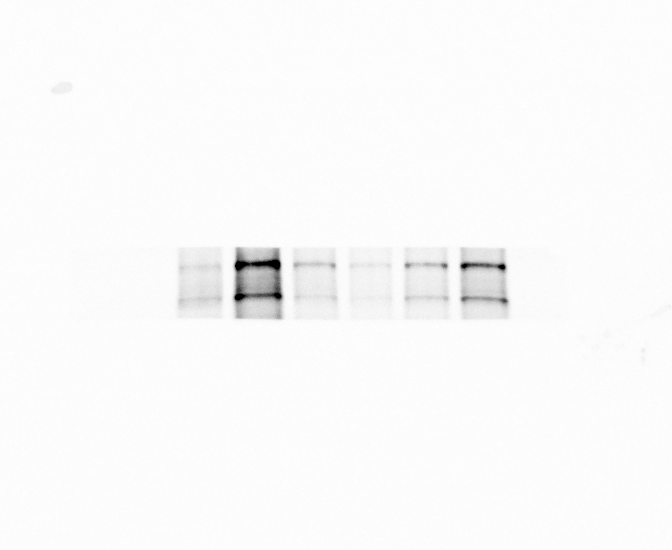

Supplement: Supplementary file 1 [file DataSheet2.zip › origin WB image/3 CIA RANKL_8bit.tif]

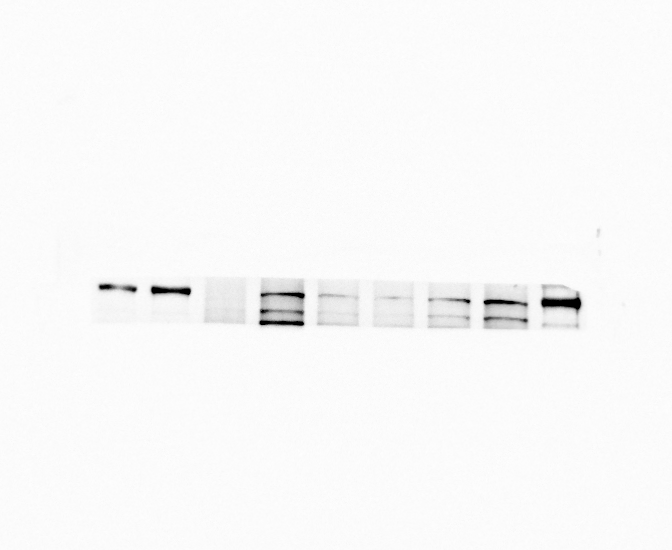

Supplement: Supplementary file 1 [file DataSheet2.zip › origin WB image/3 CIA RANK_8bit.tif]
