# Supplementary material for: Fu-zi decoction attenuate rheumatoid arthritis in vivo and in vitro by modulating RANK/RANKL signaling pathway
Source: Front Pharmacol. 2024 Jul 23;15:1423884. doi: 10.3389/fphar.2024.1423884 (PMC11300212; doi:10.3389/fphar.2024.1423884)
Supplement: Supplementary file 2 [file DataSheet1.docx]

Supplementary Material

Fu-zi decoction attenuate rheumatoid arthritis in vivo and in vitro by modulating RANK/RANKL signaling pathway

**Zhenzhen Pan^1, 2^,** **Fangchan Li^2^, Yujie Xu^1^, Huimin Ye^1^, Jiahui Liu^1^,** **Zhenhua Wang^1^, Changsheng Deng^1^, Jianping Song^1^, Manxue Mei^1^, Changqing Li^1^ ***

*** Correspondence:** Changqing Li: Email: lichq@gzucm.edu.cn

# Supplementary Figures and Tables

## Supplementary Figures


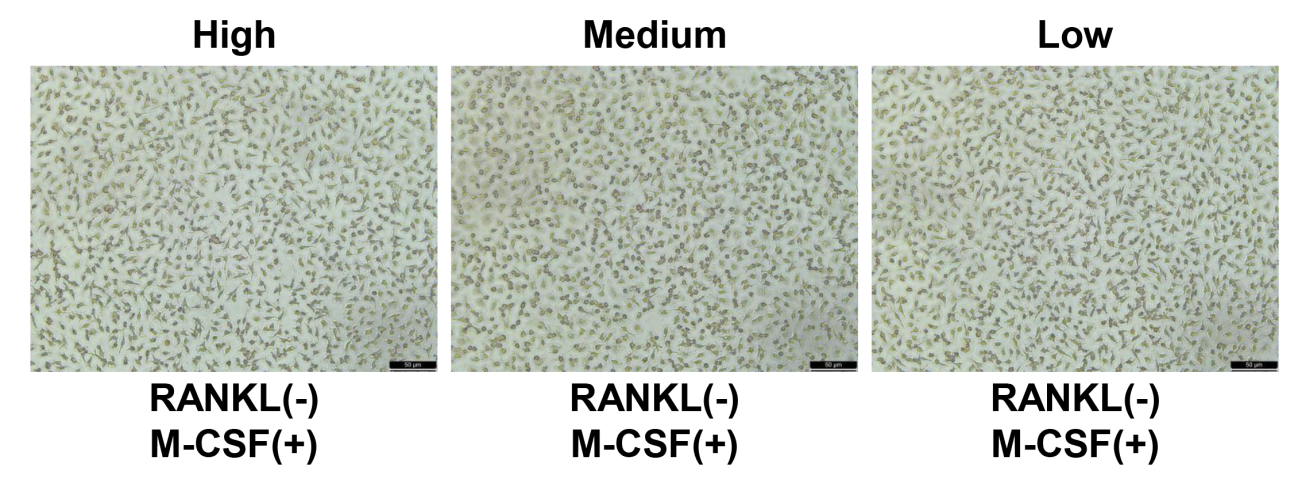


**Supplementary Figure 1** The effects of FZD treatment on BMMs
